# Intrasexual Selection for Upper Limb Length in *Homo sapiens*

**DOI:** 10.1002/ajhb.70010

**Published:** 2025-02-19

**Authors:** Neil R. Caton, David M. G. Lewis

**Affiliations:** ^1^ School of Psychology University of Queensland Brisbane Queensland Australia; ^2^ Division of Psychology Murdoch University Melbourne Western Australia Australia; ^3^ Centre for Healthy Ageing, Health Futures Institute Murdoch University Melbourne Western Australia Australia

**Keywords:** fighting ability, intrasexual selection, resource‐holding power, sexual dimorphism, upper limb length

## Abstract

**Objectives:**

Sexual selection via contest competition has equipped countless organisms with weaponry in their *appendages* to overpower their opponents. Here, we tested (1) whether greater upper limb length—measured as span controlling for biacromial width—confers an advantage in contest competition among adult humans, (2) several possible means by which upper limb length might increase success in intrasexual contest competition, and (3) whether, consistent with male–male contest competition creating stronger selection pressures than female–female contest competition, male *Homo sapiens* have greater upper limb length.

**Methods:**

We collected fight statistics and facial and body photographs from professional combatants (*N* = 715) in the Ultimate Fighting Championship (UFC; Study 1). Sexual dimorphism in upper limb length was then examined via diverse and demographically representative samples from four studies (total *N* = 6915), from Croatian adolescents and older Singaporean adults to United States Army personnel born across all major world regions (Studies 2a–2d).

**Results:**

First, we found that greater upper limb length is associated with increased success in intrasexual contest competition, an effect driven by both the capacity to grapple opponents to submission and to knock opponents unconscious (Study 1). Second, we found unequivocal, cross‐cultural evidence of unique sexual dimorphism in upper limb length *after controlling for allometry*: across four studies, men exhibited longer upper limbs than women (Studies 2a–2d).

**Conclusion:**

Upper limb length may have been shaped by intrasexual selection, with implications across the biological, anthropological, and psychological sciences.

“*But animals of all kinds*, *and their progenitors before them*, *when attacked or threatened by an enemy*, *have exerted their utmost powers in fighting and in defending themselves*.”

Charles Darwin, The *Expression* of the *Emotions* in *Man* and *Animals*

Sexual selection via contest competition has equipped countless organisms with weaponry in their *appendages* to overpower their opponents (Andersson 1994; Darwin 1872; Emlen 2008). Each of these appendage‐based weapons confers an advantage in intrasexual competition (Andersson 1994; Emlen 2008; McCullough et al. 2016). This includes enlarged claws and pincer‐like chelipeds among crustacea; horns and tusks among insects; among fish, billfish rostrums; horns, spines, and tusks among amphibians; tail clubs and cranial projections among reptiles; and, in non‐primate mammals, horns, tusks, antlers, and, in giraffes, the neck itself (Andersson 1994; Emlen 2008; McCullough et al. 2016). Male–male competition may have similarly shaped appendage‐based traits in primates. There is male‐biased sexual dimorphism in forearm length and breadth in primates (Behringer et al. 2016; Morris et al. 2019; Oishi et al. 2009; Schoonaert et al. 2007; Zihlman 1997; Zihlman and McFarland 2000). The literature on human contest competition often attributes male‐biased sexual dimorphism to male–male contest competition, interpreting it as indicative of traits shaped to increase fighting ability (Hill et al. 2017; Sell et al. 2012; Puts et al. 2015), often referred to as resource‐holding potential (or resource‐holding power, RHP; Arnott and Elwood 2009).

Human males are characterized by a similar degree of contest competition as their primate relatives (see Morris et al. 2020; but for a more thorough review, see Puts et al. 2023). Despite Darwin advancing the hypothesis that humans have evolved to increase their capacity for “striking or fighting with an enemy”^1^ a century and a half ago, limited research has examined whether human upper appendages exhibit evidence of sexual selection specifically for increasing success in contests.

To this end, the current studies test the hypothesis that the human upper limb itself has been shaped by selection specifically to increase success in real‐world agonistic exchanges. To test this hypothesis, we focused on upper limb length, which exhibits sexual dimorphism in anthropoid primates (Schoonaert et al. 2007; Zihlman 1997) and is linked to potential proxies of fighting ability in humans (e.g., greater work and peak power during deadlifting; Lockie et al. 2018). Unlike upper limb *breadth*, upper limb *length* cannot be trained (Lo et al. 2011) and does not vary with sociocultural influences (Leit et al. 2001) or post‐pubertal age (Mitchell and Lipschitz 1982). While upper limb length may be associated with throwing (hunting) power and velocity (Goranovic et al. 2023; Sharma and Mukhopadhyay 2022; Singh and Shukla 2024; Quraishi et al. 2022; but see Raschka et al. 2015), this hunting hypothesis is not mutually exclusive to the fighting hypothesis and further investigation is encouraged (for a more thorough discussion, see *Discussion*).

## 1 Hand‐to‐Hand Combat and the Evolution of Human Upper Appendages

Hand‐to‐hand combat is an ancient feature of human contest competition (Carrier and Morgan 2014; Morgan and Carrier 2013), potentially driving adaptations that increase men's RHP (Morris et al. 2020; Sell et al. 2012). Human upper appendages are the most commonly used weapon in male–male contest competition (even exceeding the use of guns, knives, and other weaponry; see Carrier and Morgan 2014) and some research suggests that these appendages have evolved to strike the most common target during agonistic exchanges: men's craniofacial skeleton (Carrier and Morgan 2014; Caton, Hannan, and Dixson 2022a; Caton, Pearson, and Dixson 2022b). Indeed, recent research—though, as an emerging body, this research is limited—suggests that there has been a four‐million‐year‐long co‐evolutionary arms race between the hominin skull and features of the hominin upper body (e.g., fist morphology: Morgan and Carrier 2013; forward arm cranking power: Morris et al. 2020). Recent—albeit limited—research suggests that hominin upper body anatomy has evolved specifically to inflict damage on the hominin skull, and the hominin skull has evolved greater robusticity to buffer the force delivered by the fists of opponents (Carrier and Morgan 2014; Morgan and Carrier 2013).

However, the evolution of human morphology reflects a complex interplay between cooperation and competition, with selective pressures shaping both prosocial behaviors and physical traits. While hominin faces have become less robust over the last few million years (Carrier and Morgan 2014; Cieri et al. 2014; Sánchez‐Villagra and Van Schaik 2019), Carrier and Morgan (2014) suggest this may be due to improved weapon technology and the decreased importance of physical strength during combat. This may reflect a larger trend toward prosocial behavior, but it should be noted that the evolution of cooperation and competition are deeply intertwined (Ito and Doebeli 2019). For example, war drives prosocial behavior (Gneezy and Fessler 2012) and the combination of in‐group favoritism with hostility toward out‐groups—known as parochial altruism (Choi and Bowles 2007)—is one of the possible explanations for the origin of human self‐domestication (Wrangham 2019). Wrangham (2019) expands on these explanations by proposing that self‐domestication in humans, characterized by reduced *reactive* aggression, may have been influenced by the use of lethal weapons and the capacity for language‐based conspiracy, which allowed less physically dominant individuals to cooperatively eliminate more dominant males.

Crucially, the emergence of weapons and the accompanying reduction in the importance of physical formidability do not diminish the evolutionary significance of hand‐to‐hand combat but instead underscore its foundational role in shaping human morphology and behavior. The selective pressure posed by powerful individuals may have indeed driven the development of these psychological systems, such as those underpinning language‐based conspiracy, and traits evolved to counteract competitors' morphological advantages. This highlights that while self‐domestication reflects a shift in selective pressures, at least for in‐group counterparts, it does not negate—but underscores—the role of contest competition in human evolution (also see Caton et al. 2024). Yet this area of investigation has only recently emerged, with limited research demonstrating links between features of the upper appendages and real‐world fighting success.^4^

Prior research has relied either on laboratory studies (e.g., Morris et al. 2020; Morgan and Carrier 2013), examined both upper limb length and biacromial width in a single variable (e.g., Dixson et al. 2017; Richardson 2021), or both. Evolutionary biologists emphasize that while sexual dimorphism can indicate a trait is shaped by sexual selection, there should also be a direct connection between the trait and success in mating competition (Hodges‐Simeon et al. 2021). To test this relationship, real‐world contest data is essential, as it accounts for the possibility that opponents in real‐world contests will also possess morphological structures (e.g., robust craniofacial morphology; Carrier and Morgan 2014) or psychological systems (e.g., fighting skill; Briffa and Lane 2017; Caton and Dixson 2022a; Caton, Hannan, and Dixson 2022a; Lane and Briffa 2020) evolved to overcome their competitor's morphological advantage. Humans may possess craniofacial morphology which buffers the damage delivered by the fists of opponents (Carrier and Morgan 2014) or perform more skilled fighting maneuvers to increase their likelihood of victory against a larger competitor (Briffa and Lane 2017; Caton, Hannan, and Dixson 2022a). Contest data offers a more comprehensive framework for understanding RHP by considering the dynamic interplay of morphological and psychological traits in competitive environments.

Among research that has examined real‐world fighting success, arguably, the upper limbs themselves may not have been directly examined. For example, Dixson et al. (2017) and Richardson (2021) examined arm *span*, measured from fingertip to fingertip.^2^ ^,^ ^3^ ^,^ ^4^ These studies have not uniquely measured upper limb length; span is the sum of biacromial (i.e., shoulder) width and upper limb length, and therefore conflates them. Biacromial width predicts fighting performance (Katić et al. 2005) and has long been theorized to increase success in male–male contest competition (Puts 2010; Sell et al. 2012; Zilioli et al. 2015); any relationship between *span* and fighting success could be derived from biacromial width, not upper limb length.

Another key shortcoming of prior research is that it has failed to account for lower limb length (e.g., Dixson et al. 2017; Richardson 2021). Greater lower limb length is theorized to confer advantages in intrasexual competition (e.g., ground combat, grappling, leg sweep, roundhouse kick: Richardson 2021; movement speed: Bramble and Lieberman 2004). Greater lower limb length is also correlated with greater stature (Moshkdanian et al. 2014; Nor et al. 2013) and Carrier (2011) suggested that greater energy can be delivered on an opponent in downward versus upward directed strikes, providing taller individuals with a competitive advantage. Upper limb length could therefore be a by‐product of an allometric system that increases overall limb length, not a reflection of sexual selection. Without independently accounting for other morphological features such as biacromial width and lower limb length, it is not possible to determine whether upper limb length is a true predictor of success in agonistic exchanges.

With the above in mind, we aimed to examine Darwin's (1872) hypothesis that the human upper limbs, specifically upper limb *length*, may have been influenced by contest competition. To examine this, we measured and then controlled for biacromial width to measure the unique effect of upper limb length.^4^

## 2 Upper Limb Length and RHP: Three Distinct Hypotheses

Morphological weapons can increase the chances of victory without necessarily causing physical harm; some weapons serve as signals (McCullough et al. 2016), while others deter conspecifics from resources without inflicting injury (Andersson 1994). This highlights the need for human contest competition research to extend beyond simple measures of overall victory (e.g., Caton, Pearson, and Dixson 2022b; Dixson et al. 2017; Richardson 2021; Zilioli et al. 2015). There are at least three potential means by which greater upper limb length could confer an advantage in hand‐to‐hand combat.

Hypothesis 1. The distance hypothesis.

Sexual selection has shaped animal morphology to enhance specific fighting styles and maneuvers (McCullough et al. 2014). Richardson (2021) discussed but did not empirically test that upper limb length might have afforded greater distance between the animal and their opponent.^2^ ^,^ ^3^ Because animals with longer upper limbs were better able to position themselves within striking distance but keep their opponent outside the range of retaliation, they were better able to: (1) land strikes on their opponent; and (2) evade attacks (strikes, takedowns) from their opponent. However, no research has examined upper limb length (or span) in relation to striking or defensive abilities. Potentially, upper limb length increases RHP through: (1) increased striking accuracy; and/or (2) increased evasion abilities. We refer to this as the distance hypothesis.

Hypothesis 2. The grappler hypothesis.

Hand‐to‐hand combat in the form of grappling and wrestling is well‐documented across hunter‐gatherer groups (Moreno 2011). Mirzaei et al. (2013) and Erawan et al. (2020) speculated that upper limb length might increase an organism's RHP through the successful execution of grappling strategies (e.g., reverse lift, back arch, headlock, body destabilization). Potentially, upper limb length increases RHP due to increased grappling abilities. However, no research has examined upper limb length (or span) in relation to grappling or submission abilities.

Successful grappling abilities may ultimately eventuate in a submission, noted to bear similarities to retreats in animal contests (Caton and Dixson 2022a; Lane and Briffa 2020). Submission victories involve an arduous process requiring continual efforts to grapple and lock an opponent, often terminating on the ground (Caton and Dixson 2022a). Grappling abilities are specific abilities (e.g., takedown) whereas a submission represents the ultimate result of a long series of standing, clinch, and ground‐based grappling abilities (Caton and Dixson 2022a). Submission victories might more holistically capture a range of grappling abilities not reflected within existing grappling measures (i.e., grappling accuracy, measured as the fighter's total takedowns landed divided by the fighter's total takedowns attempted). While upper limb length may not be associated with grappling accuracy specifically then, upper limb length may be associated with submission victories more broadly. We refer to this as the *grappler hypothesis*.

Hypothesis 3. The knockout hypothesis.

Larger human male morphology is theorized to have evolved to exert greater damage on conspecifics (Caton, Hannan, and Dixson 2022a; Caton, Pearson, and Dixson 2022b; Caton et al. 2023; Puts 2010; Sell et al. 2012; Sell et al. 2016; Sell et al. 2017; Zilioli et al. 2015). Male‐biased sexual dimorphism in biacromial width, bone density, bone strength, and body mass are theorized to have evolved to exert greater damage on opponents during contests (Puts 2010; Sell et al. 2012). Yet, no research has discovered an association between upper limb length (or span or biacromial width) and knockout victories in contest competition.^2,3,4^ Here, we empirically examined the hypothesis—the knockout hypothesis—that upper limb length evolved specifically to increase RHP during agonistic exchanges through increased knockout success.

Upper limb length can influence the mechanics of a strike, potentially impacting knockout success. Although limb length itself does not directly affect force output—since force output is a function of the force synergistic muscles produce and the mechanical advantage through which the muscles act (for further reading, see Finni et al. 2023; Herzog 2017; Lieber and Ward 2011; Maas and Sandercock 2010) and peak forces were not greater in individuals with longer upper limbs when deadlifting a given weight (Lockie et al. 2018)—longer upper limbs can alter how force is applied and the resulting work done. With work as the product of force and displacement (Parke 2020; Zatsiorsky and Prilutsky 2012), longer upper limbs could increase the distance over which force is applied, producing more work on an opponent. With power as force times velocity (Parke 2020; Zatsiorsky and Prilutsky 2012), longer upper limbs may allow the force to be applied at a higher velocity, producing greater striking power. Lever mechanics supports this basic idea—where lever length is regarded as a proxy for upper limb length—where longer levers are afforded increased distance and allow force to be applied at a higher velocity, improving performance across a range of sports (Ackland et al. 2009; also see *Discussion*, for research on upper limb length and velocity in throwing sports). For striking in boxing, karate, and taekwondo specifically, Ackland et al. (2009) theoretically noted that the linear velocity of the end point is in part determined by the length of the lever.

While direct studies linking upper limb length to work and power are limited, swimming performance research suggests that span and upper limb length are strong predictors of swimming performance time (Lätt et al. 2010; Rozi et al. 2018). This research notes that the length of the upper limbs may be associated with biomechanical factors relevant to propulsion (Lätt et al. 2010; Rozi et al. 2018). Fett et al. (2020) noted that greater span in tennis players provides the opportunity to transfer greater velocity to the racquet head, finding that greater span predicted increased serve velocity. Most directly related to upper limb length and work and peak power, Lockie et al. (2018) found that individuals with longer upper limbs produced greater work and peak power in the context of deadlifting.

Taken together, longer upper limbs in humans may contribute to knockout success. Given that knockouts involve the complete loss of consciousness, this incapacitation could have increased the likelihood of fatal outcomes in evolutionary contexts: a single, powerful strike to an opponent's head can potentially lead to death (Fenton et al. 2003; Flynn et al. 2016). If the opponent was not instantly killed, a knockout would have left the opponent vulnerable, providing an opportunity for a potentially fatal follow‐up action (Flynn et al. 2016; Hånell and Rostami 2020).

### 2.1 Upper Limb Length and Sexual Dimorphism

If male–male combat drove the evolution of male‐biased sexual dimorphism in traits that increase RHP (Morris et al. 2020; for more thorough reviews, see Hill et al. 2017; Puts 2010; Puts et al. 2015; Puts et al. 2023), *Homo sapiens* upper limb length, if shaped by intrasexual selection, should exhibit sexual dimorphism (Andersson 1994; Darwin 1872; Emlen 2008; Hill et al. 2017; Puts 2010). Indeed, sexual dimorphism in human morphology has been used as the sole evidentiary criterion for establishing whether a human morphological feature was shaped by contest competition (Andersson 1994; Darwin 1872; Emlen 2008; Hill et al. 2017; Puts 2010; for an explicit empirical example, see Morris et al. 2020).

However, previous research has examined span and failed to disentangle the currently unknown sexual dimorphism in upper limb length from the previously known sexual dimorphism in biacromial width (Aggarwal et al. 2000; Bjelica et al. 2012; Floyd 2017; Richardson 2021; Quanjer et al. 2014). When research has examined upper limb length (e.g., Živičnjak et al. 2003), they have not accounted for the previously known sexual dimorphisms in weight, height, and/or lower limb length (Bertamini and Bennett 2009; Ruff 1987; Sell et al. 2012). Without accounting for these allometric components, upper limb length could be a by‐product of an overarching allometric system, not a reflection of sexual selection.

### 2.2 The Present Work

The present work aimed to test aspects of the hypothesis that upper limb length has been shaped by sexual selection in humans (e.g., other morphological features proposed to have been shaped by sexual selection include facial width‐to‐height ratio; Carré and McCormick 2008, and waist‐to‐hip ratio; Singh 1993). In line with previous guidelines (Andersson 1994), we focused on two evidentiary criteria for establishing a unique characteristic shaped by sexual selection: (1) increased success in mating competition after accounting for allometry; and (2) sexual dimorphism after accounting for allometry, at least during the periods of mating competition. Because characteristics uniquely shaped by sexual selection are associated with an array of behavioral, psychological, and morphological traits, the identification of such characteristics is important for numerous domains across the biological, anthropological, and psychological sciences (Andersson 1994; Puts 2010).

Recent recommendations highlight the potential of sports performance data to address evolutionary questions (Wilson et al. 2017). While not all sports data are equally suitable, UFC data is considered a valuable ‘quasi‐Darwinian’ (Zilioli et al. 2015) proxy for human contest competition due to its minimal regulations (e.g., strikes to the eyes and genitals are forbidden) and highly aggressive nature (Dixson et al. 2017; Schild and Zettler 2021; Zilioli et al. 2015). However, previous research emphasizes the need for caution in interpreting analyses based on UFC data, as its limitations depend on the specific context of each study (Caton, Hannan, and Dixson 2022a; Caton, Pearson, and Dixson 2022b; Caton et al. 2024). For instance, the UFC's weight categories constrain body size variation, complicating its relationship with fighting performance (see Caton et al. 2024) while features like height, lower limb length, upper limb length retain variation within these categories. More generally, UFC combatants may exhibit extreme muscle mass and training patterns, potentially reducing the true advantage of static morphological features. Previous research has noted that alternative populations without weight constraints and with lower skill levels may yield larger effect sizes (Caton, Hannan, and Dixson 2022a; Caton, Pearson, and Dixson 2022b) which appears to be the case (Caton et al. 2024), as estimates in this field are often small (Caton, Hannan, and Dixson 2022a; Richardson 2021; Třebický et al. 2013; Třebický et al. 2015; Zilioli et al. 2015). Despite these nuances, UFC data remains indispensable for testing hypotheses about human contest competition—an area of research still in its early stages—as it collects a unique and large combination of fight performance (e.g., knockout, striking abilities), anthropometric, and photograph‐derived data across a large sample size, which is required to detect reliable effects in this research (Caton, Hannan, and Dixson 2022a; Caton, Pearson, and Dixson 2022b). For the present work, UFC data is the only means by which the current hypotheses can currently be examined.

Consequently, Study 1 used real‐world contest data to assess if individuals with greater upper limb length experienced greater RHP during agonistic exchanges. Even after controlling for other morphological features (e.g., weight, height, lower limb length, biacromial width) and other important covariates (e.g., fighter's age, debut date), we anticipated that individuals with greater upper limb length would experience greater fighting success (H_1_) through six mediators: for the distance hypothesis, striking accuracy, striking defense, and grappling defense (H_2_); for the grappler hypothesis, grappling accuracy and submission victories (H_3_); and for the knockout hypothesis, knockout victories (H_4_).

Following on from Study 1, Study 2 tested the hypothesized sexual dimorphism in upper limb length across a diverse combination of samples, after accounting for allometry. This included mixed‐martial‐artists (Study 2a), in people young (Croatian adolescents; Study 2b) and old (older Singaporean adults; Study 2c), and over 6000 US Army personnel (Study 2d), a uniquely population‐representative sample.

## 3 Materials and Methods

### 3.1 Study 1: Upper Limb Length and Fighting Performance

In line with previous research on human contest competition (e.g., Aung et al. 2021; Caton, Hannan, and Dixson 2022a; Caton, Pearson, and Dixson 2022b; Zilioli et al. 2015), fight statistics and photographs were collected for all fighters on ufc.com (*N* = 715). Demographic data were collected for fighter's age, professional debut date, span, lower limb length, weight, height, and fighter's sex (113 females, 602 males; see ESM for all descriptive statistics). We direct readers to the Supporting Information for more detail on these variables, including lower limb length.

Previous research (e.g., Quanjer et al. 2014; Richardson 2021) has used span was divided by height as a metric of relative upper limb length; however, ratio variables can be problematic statistically (for a detailed discussion, see Caton and Dixson 2022b; Nakagawa et al. 2017). We therefore followed recommendations to avoid anatomical ratio measures and instead control for the allometric variable, modeled as a covariate (for a detailed discussion, see Caton and Dixson 2022b; Nakagawa et al. 2017). We therefore modeled span as a predictor, controlling for height, rather than span divided by height. It should be noted that all results were initially conducted with span divided by height and the significance of the results does not change depending on the method used, but it is nonetheless preferable to avoid anatomical ratio measurements to yield more reliable and interpretable coefficients both for the individual study and the broader literature at hand (Caton and Dixson 2022b; Nakagawa et al. 2017).

We further collected data on total wins, losses, and draws, which we summed to compute a “total fights” variable. This excluded disqualifications and *no contests* because, in these very rare cases, a fighter was disqualified or a fight was terminated meaning that no actual outcome (win or loss) was reached. In line with prior research (Aung et al. 2021; Caton, Hannan, and Dixson 2022a; Caton, Pearson, and Dixson 2022b; Zilioli et al. 2015), fighting success was then calculated as total wins divided by total fights. Detailed information on the nature of wins and losses (i.e., by knockout or technical knockout [KO/TKO], submission, decision) were collected from espn.com. Where possible, all data were cross‐referenced between ufc.com and espn.com for accuracy, with preference toward espn.com for win/loss statistics because it is a frequently updated major sports network with more detailed information (e.g., specific fight information, dates on which fights occurred). Data were entered by two research assistants from 15 May 2019 to 6 April 2020, who recorded the date on which data were entered for each fighter. This allowed the data to be independently confirmed by two additional research assistants.

UFC facial photographs have been advantageous for previous research because fighters are similarly postured and positioned at approximately similar distances from the camera (Caton, Hannan, and Dixson 2022a; Caton, Pearson, and Dixson 2022b; Zilioli et al. 2015). Like previous research on facial morphology and fighting success (Caton, Hannan, and Dixson 2022a; Caton, Pearson, and Dixson 2022b), research assistants landmarked the top of each acromion in the entire collection of 715 faces from ufc.com (i.e., approximately 180 faces per research assistant) in tpsDig2 (version 2.31; Rohlf 2018) to measure biacromial width (for more detailed information, see ESM). Landmarking is a widely‐used technique in the biological sciences that is used to describe the length, width, and/or shape of morphological traits, where individual landmarks are used to capture anatomically similar positions in different stimuli (Adams and Otárola‐Castillo 2013; Bookstein 1997; Caton, Pearson, and Dixson 2022b). We computed the distance between these two acromion landmarks to create a key variable not included in previous contest competition research: biacromial width. Existing data only records span, which inherently captures the sum of biacromial width and upper limb length but fails to disentangle these fundamentally different variables.

Consequently, biacromial width is scaled using photograph‐based measurements, where the photographs themselves are approximately standardized. Deriving morphological measurements from UFC photographs is a widely used method in human contest competition research (e.g., Zilioli et al. 2015; Třebický et al. 2013, 2015; Caton, Hannan, and Dixson 2022a; Caton, Pearson, and Dixson 2022b). While we standardized images in the sense that the images are equally sized at 350 × 254 pixels, the photographs themselves are approximately standardized (e.g., in that there is approximately similar lighting, background, neutral expression, and distance from the camera). We assume that the same camera and setup is *not* used for all individuals examined but infer from the photographs themselves, and the convergent associations, that this is approximate. Biacromial width derived from these images showed convergent validity, correlating positively with other morphological traits (e.g., span, height, leg length, and weight), knockout success and male‐biased sexual dimorphism (Table 1). Although ideal measurements would involve direct anthropometric data, photograph‐derived measurements are the only available method for testing these hypotheses in real‐world contest data, which we acknowledge as a limitation.

**TABLE 1:** Correlation matrix.

	1	2	3	4	5	6	7	8	9	10	11	12	13	14	15	16	17
1. Span to height^d^	—																
2. Biacromial width	0.21^a^	—															
3. Leg length	0.22^a^	0.12^b^	—														
4. Height	0.13^a^	0.17^a^	0.75^a^	—													
5. Weight	0.24^a^	0.21^a^	0.65^a^	0.79^a^	—												
6. Age	0.12^b^	0.02	0.14^a^	0.10^b^	0.19^a^	—											
7. Debut date	−0.08^c^	−0.02	−0.12^b^	−0.10^b^	−0.10^b^	−0.66^a^	—										
8. Sex	0.26^a^	0.24^a^	0.35^a^	0.53^a^	0.45^a^	0.10^b^	−0.18^a^	—									
9. KO/TKO wins	0.16^a^	0.10^b^	0.21^a^	0.26^a^	0.29^a^	0.28^a^	−0.35^a^	0.28^a^	—								
10. Submission wins	0.06	−0.04	0.06	0.08^c^	0.01	0.22^a^	−0.30^a^	0.17^a^	−0.05	—							
11. Decision wins	0.00	−0.07	−0.12^b^	−0.20^a^	−0.18^a^	0.32^a^	−0.44^a^	0.05	0.13^a^	0.11^b^	—						
12. Striking accuracy	0.05	0.03	0.17^a^	0.19^a^	0.25^a^	0.00	0.04	0.02	0.15^a^	−0.04	−0.13^a^	—					
13. Strike defense	0.02	−0.04	−0.06	−0.15^a^	−0.19^a^	0.04	−0.14^a^	0.01	0.06	−0.06	0.29^a^	−0.04	—				
14. Takedown defense	0.05	0.01	0.06	0.05	0.09^c^	−0.10^c^	0.06	0.05	0.07	−0.21^a^	0.10^b^	0.13^b^	0.24^a^	—			
15. Grappling accuracy	0.04	−0.05	0.11^b^	0.03	0.03	−0.02	0.00	−0.08	0.02	−0.07	−0.03	0.18^a^	0.11^c^	0.12^b^	—		
16. Fighting success	0.09^c^	0.05	0.05	0.06	0.07	−0.32^a^	0.31^a^	0.11^b^	0.03	−0.04	−0.04	0.16^a^	0.07	0.18^a^	0.03	—	
17. Span (non‐ratio)^d^	0.55^a^	0.25^a^	0.73^a^	0.90^a^	0.78^a^	0.15^a^	−0.13^a^	0.55^a^	0.29^a^	0.10^c^	−0.18^a^	0.19^a^	−0.11^b^	0.05	0.05	0.09^c^	—

^a^ *p* < 0.001.

^b^ *p* < 0.01.

^c^ *p* < 0.05.

^d^ This is a bivariate correlation and thus we refer to this variable as span. We only refer to span as upper limb length when examining the coefficient of span in a model that controls for biacromial width.

We disentangled the effect of biacromial width from span by examining span in a model that controls for biacromial width; this allowed us to measure the unique effect of upper limb length in our analyses. To elaborate, by examining the effect of span in a model that controls for biacromial width, we are by definition able to examine the effect of span with biacromial width *held constant*. This means that, in a multiple regression model or partial correlation model that includes both span and biacromial width, any coefficient for span now reflects the unique effect of upper limb length. For this reason, in Studies 1 and 2a, we refer to span when controlling for biacromial width as upper limb length. When we do not control for biacromial width in the same model as span, such as in a bivariate correlation, we refer to this as span and not as upper limb length.

Finally, data on fighter's lifetime (1) striking accuracy, (2) strike defense, (3) grappling defense, and (4) grappling accuracy were collected from ufc.com. Striking accuracy is calculated by the total strikes landed divided by the total strikes attempted. Strike defense is calculated by the fighter's opponent's total strikes landed divided by the fighter's opponent's total strikes attempted. Grappling defense is calculated by the fighter's opponent's total takedowns landed divided by the fighter's opponent's total takedowns attempted. Grappling accuracy is calculated by the fighter's total takedowns landed divided by the fighter's total takedowns attempted (for detailed definitions, see James et al. 2017). The UFC (a billion‐dollar organization) is the only organization to collect this fight‐specific performance data, and across a very large sample of contests. This would ordinarily be extremely expensive to collect through other sampling methods (e.g., simple random sampling) and human contest research requires large sample sizes to detect effects, should they exist (Caton, Hannan, and Dixson 2022a; Caton and Dixson 2022a; Richardson and Gilman 2019). The dataset for Study 1 is available on the Open Science Framework (https://osf.io/v3qpn/).

### 3.2 Study 2: Sexual Dimorphism in Upper Limb Length

#### 3.2.1 Study 2a: UFC Fighters

Study 2a comprised the same sample (715 fighters: 113 females, 602 males) and variables as Study 1, including sex, span, biacromial width, lower limb length, weight, height, age, and fighters' professional debut date. Biacromial width was controlled for to analyze the unique effects of upper limb length. Like Study 1, in Study 2a, span with biacromial width controlled for is referred to as upper limb length. Studies 1 and 2a are the only studies in the present work which measure upper limb length as span controlling for biacromial width. Because Studies 2b–2d do not statistically entangle both upper limb length and biacromial width in a single span variable, there is no need to statistically disentangle biacromial width from span to measure upper limb length in Studies 2b–2d.

#### 3.2.2 Study 2b: Croatian Adolescents

Study 2b re‐analyzed data from Živičnjak et al. (2003, tables 2–4), who collected data from 5155 Croatian children and adolescents (2591 females, 2564 males) aged 3–18 years. This data is collapsed across sex and age groups, meaning that data exists for 32 cases (i.e., 16 age groups across two sexes). Živičnjak et al. (2003) collected data on upper limb length (measured as total upper limb length; that is, fingertip to acromion), height, and lower limb length (measured as the height of anterior superior iliac spine). Length‐based measurements were quantified in centimeters; therefore, unstandardized coefficients from multiple regressions represent the rate at which upper limb length increases in males over females (e.g., a *B* coefficient of 0.21 represents that males' upper limb length is 0.21 cm greater than females, controlling for other variables in the model).

#### 3.2.3 Study 2c: Older Singaporean Adults

Study 2c used a publicly available dataset from Lee et al. (2020) who collected anthropometric measurements from 100 older Singaporean adults (50 male, 50 female) aged 60 to 93 years (*M* = 74.14, SD = 8.62). Lee et al. (2020) collected data on stature (also known as height; *M*_centimetres_ = 160.68, SD_centimetres_ = 6.39), upper limb length (*M*_centimetres_ = 75.66, SD_centimetres_ = 4.08; measured as the combined distance from the acromion to the elbow [shoulder‐to‐elbow length] and from the elbow to the fingertip [elbow‐to‐fingertip length]), and lower limb length (*M*_centimetres_ = 93.21, SD_centimetres_ = 4.67; measured as the distance from the foot to the hip, or the height of the anterior superior iliac spine, also known as standing hip height). As above, length‐based measurements were quantified in centimeters; therefore, unstandardized coefficients from multiple regressions represent the rate at which upper limb length increases in males over females.

#### 3.2.4 Study 2d: US Army Personnel

Study 2d used the ANSUR (Anthropometric Study of US Army Personnel) II publicly available dataset (Paquette 2009) which includes 6068 adult US army personnel (4082 men, coded as “1”; *M*_age_ = 30.16 ± 8.81; 1986 women, coded as “0”; *M*_age_ = 28.94 ± 8.33). The ANSUR II dataset is population‐representative; the US Army personnel in this dataset, which include reservists, are demographically representative of the overall population (Caton and Dixson 2022b) and engage in over 250 distinct professions, ranging from musicians and public affairs specialists to plumbers and dental specialists (Paquette 2009).

The ANSUR II dataset collected data on 93 anthropometric measurements, including stature (also known as height; *M*_centimetres_ = 171.44, SD_centimetres_ = 9.00), weight (in kilograms; *M* = 79.71; SD = 15.65), upper limb length (*M*_centimetres_ = 82.11, SD_centimetres_ = 5.08; measured as the combined distance from the acromion to the elbow [shoulder‐to‐elbow length] and from the elbow to the fingertip, called forearm‐hand length in this dataset), and lower limb length (*M*_centimetres_ = 110.21, SD_centimetres_ = 6.85; measured as the straight‐line distance between the plane of the bottom of the right foot to the trochanter landmark). As above, length‐based measurements were quantified in centimeters with unstandardized coefficients from multiple regressions representing the rate at which upper limb length is greater in males over females.

ANSUR II also collected data on the subjects' birth location, with participants born across Africa (*n* = 35), Europe (*n* = 147), Asia (*n* = 146), Oceania (*n* = 54), and North (*n* = 5388), Central (*n* = 254), and South America (*n* = 44) (see ESM for information on which countries were grouped into which world regions). Datasets for Studies 2a–2d are available on the Open Science Framework (https://osf.io/v3qpn/).

## 4 Results

### 4.1 Study 1: Upper Limb Length and Fighting Performance

Bivariate correlations were run between span, biacromial width, lower limb length, weight, height, age, debut date, sex, KO/TKO wins (knockout success), submission wins, decision wins, KO/TKO losses, submission losses, decision losses, striking accuracy, strike defense, takedown defense, and grappling accuracy (Table 1). Notably, there were bivariate associations between span and knockout success, biacromial width and knockout success, and leg length and knockout success (see Table 1), suggesting the need to disentangle these related variables.

All regression‐based models in the present work (Studies 1 and 2a–2d) present unstandardized coefficients to allow for a more meaningful interpretation. For the following regressions, we report unstandardized coefficients to allow for a scientifically and biologically meaningful interpretation of upper limb length on fighting‐oriented variables, where the unstandardized coefficient represents the rate at which the outcome variable (e.g., fighting success) increases with every one‐centimeter increase in upper limb length. We report this in centimeters to allow for a meaningful and interpretative understanding of the unstandardized coefficients; we deemed a millimeter conversion to be too small for an immediate interpretation of the unstandardized coefficient.

First, we tested our main hypothesis of interest, the knockout hypothesis, that upper limb length increases fighting success through knockout success. A mediation analysis (SPSS PROCESS macro; model 4; v.4.1; 10 000 bootstrap samples; Hayes 2013) was run for the relation between upper limb length and fighting success as mediated by knockout success, controlling for biacromial width, weight, height, lower limb length, age, debut date, total fights, and sex. When measuring knockout victories, we also controlled for submission and decision victories, as these are statistically related (but theoretically distinct) avenues to victory (Caton and Dixson 2022a; Lane and Briffa 2020). This also allows us to adjust for fighters' general ability to win via other methods, and thus examine the unique variance associated with knockout wins specifically. These analyses differed to the analyses for distance and grappler hypotheses in this respect because these analyses do not examine a specific avenue to victory or defeat.

**FIGURE 1:**
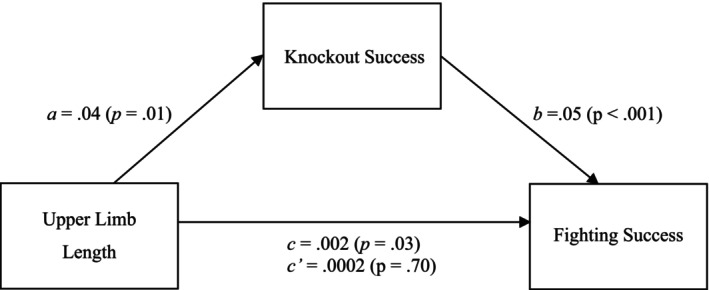
The knockout hypothesis: Upper limb length increases fighting success through knockout success. Unstandardized regression coefficients (with *p*‐values in brackets) for the relationship between upper limb length and fighting success as mediated by knockout success. Biacromial width, lower limb length, height, weight, age, debut date, total fights, sex, and wins by submission and decision were included as covariates but are not included in the figure. Indirect (a*b), direct (c’) and total effects (c) can be found in the figure.

Results supported the hypothesis that upper limb length was associated with fighting success (H_1_; Figure 1). Results of the bias‐corrected bootstrapped analyses further supported the knockout hypothesis (H_4_): upper limb length exhibited a significant indirect effect on fighting success via knockout success (*ab* path = 0.002, bootstrap SE = 0.001, 95% CI [0.0002, 0.004]).

There was no support for the distance hypothesis (striking accuracy, striking defense, or grappling defense; H_2_) or the grappler hypothesis in relation to grappling accuracy (H_3_). These variables were not significantly related to upper limb length and are reported in the ESM. While upper limb length did not predict grappling accuracy, this does not mean that upper limb length is not associated with submission victories, which is the ultimate outcome of successful grappling.

**FIGURE 2:**
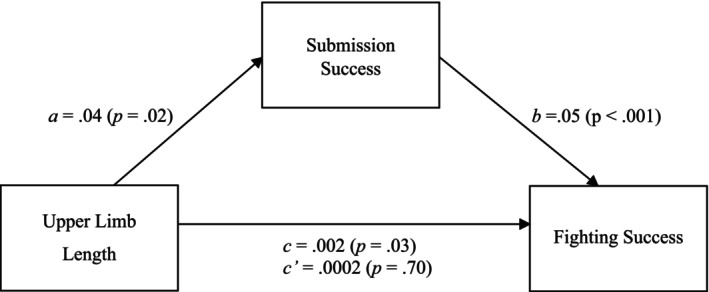
The grappler hypothesis: Upper limb length increases fighting success via submission success. Unstandardized regression coefficients (with *p*‐values in brackets) for the relationship between upper limb length and fighting success as mediated by submission success. Biacromial width, lower limb length, height, weight, age, debut date, total fights, sex, and wins by KO/TKO and decision were included as covariates but are not included in the figure. Indirect (a*b), direct (c’) and total effects (c) can be found in the figure.

Consequently, a mediation analysis (SPSS PROCESS macro; model 4; v.4.1; 10 000 bootstrap samples; Hayes 2013) was conducted to examine the relation between upper limb length and fighting success, with submission success as the mediator. We controlled for biacromial width, weight, height, lower limb length, age, debut date, total fights, sex, and knockout and decision victories. The results of the bias‐corrected bootstrapped analyses supported the grappler hypothesis in regard to submission victories (H_3_; Figure 2): upper limb length exhibited a significant indirect effect on fighting success via submission success (*ab* path = 0.002, bootstrap SE = 0.001, 95% CI [0.00005, 0.004]).

We also conducted all analyses but (1) not controlling for biacromial width and (2) using biacromial width as the focal predictor variable. To summarize the analyses without controlling for biacromial width, most analyses remained the same without including biacromial width. This finding supports previous research, suggesting that analyses controlling for height (Dixson et al. 2017; Richardson 2021) or weight (Richardson 2021) may have captured the effects of upper limb length, thereby increasing confidence that these studies may indeed reflect the influence of upper limb length. To summarize the biacromial width analyses, all associations between biacromial width and those variables related to contest competition were non‐significant. We note that this does not mean that biacromial width was not an important variable for inclusion in the models focused on upper limb length, as biacromial width exhibited positive bivariate associations with other morphological features (e.g., weight, height, leg length, span) and knockout success (see Table 1) and its removal affected the significance of the indirect effect (but not the individual direct associations) of span on fighting success via submission success. We direct readers to the Supporting Information for a more thorough discussion on the importance of including biacromial width in the main models.

### 4.2 Study 2: Sexual Dimorphism in Upper Limb Length

Partial correlations were initially conducted for all following results, but it was decided that multiple regressions would instead be used for all the following analyses for consistency in statistical analyses across all studies in the present work. It should be noted that the statistical significance of all following results did not change depending on the statistical method used. Given that the below analyses are multiple regression analyses, sex must be modeled as a predictor of upper limb length (the outcome variable) to be suitable for multiple regression analyses, controlling for the other variables modeled as covariates (e.g., height, lower limb length). For the absolute and allometric sexual dimorphism for each of the below populations, refer to Table S1 in the Supporting Information.

#### 4.2.1 Study 2a: UFC Fighters

To examine the sexual dimorphism in upper limb length in UFC fighters, a multiple regression was run between sex and upper limb length, controlling for weight, height, biacromial width, age, lower limb length, and the fighters' debut date. This analysis revealed precisely the hypothesized sex difference: men's upper limb length (controlling for their body size, as captured by the control variables above) was significantly greater than women's, *B* = 2.80, *t*(548) = 4.31, *p* < 0.001.

#### 4.2.2 Study 2b: Croatian Adolescents

We ran a multiple regression among the data from the Croatian adolescents, between sex and upper limb length, controlling for the available variables of age, height, and lower limb length. The observed sex difference in upper limb length replicated in this sample, *B* = 0.38, *t*(27) = 4.56, *p* < 0.001; among children and adolescents aged 3 to 18, boys have longer upper limb length than girls.

#### 4.2.3 Study 2c: Older Singaporean Adults

The sex difference in upper limb length was replicated in the sample of older Singaporean adults as well. After controlling for age, height, and lower limb length, men's upper limb length was greater than women's, *B* = 2.08, *t*(95) = 3.40, *p* = 0.001.

#### 4.2.4 Study 2d: US Army Personnel

The sexual dimorphism replicated yet again in the diverse and demographically representative sample of US army personnel. After controlling for age, height, weight, and lower limb length, men's upper limb length was greater than women's, *B* = 0.78, *t*(6062) = 10.78, *p* < 0.001.

## 5 Discussion

Intrasexual selection has equipped organisms across diverse taxa with appendage‐based weaponry (Andersson 1994; Darwin 1872; Emlen 2008). The current studies provide several key forms of evidence in support of the hypothesis that the upper limbs of *Homo sapiens* —a species with a long evolutionary history of hand‐to‐hand combat (Carrier and Morgan 2014)*—*evolved to increase fighting ability in hand‐to‐hand combat.

Study 1 presented analyses showing that greater upper limb *length* in *Homo sapiens* predicts real‐world fighting success. Moreover, we tested multiple different means by which this relationship between greater upper limb length (after controlling for allometry through manifold other variables) and fighting success could occur. This included advancing and testing several distinct hypotheses, finding a pattern of results which suggested that selection favored greater upper limb length because it increases the capacity to both grapple an opponent to submission and knock an opponent unconscious.

Specifically, Study 1 found support for the knockout hypothesis: upper limb length contributes to fighting success (H_1_) through knockout success (H_4_) but not through striking accuracy, striking defense, or takedown defense (H_2_; the distance hypothesis) or grappling accuracy (H_3_; the grappler hypothesis). Further, there was also support for the grappler hypothesis in relation to submission victories: upper limb length contributes to fighting success through submission success. These findings represent the first empirical support for the association between knockout and submission victories and the length of the human upper appendages (or span). The findings of the grappler hypothesis highlight a distinction between grappling accuracy (which involves, while standing, launching an opponent into the air to then be thrown onto the ground) and submission victories (which involves an arduous process requiring continual efforts to grapple and lock an opponent whilst on the ground). This distinction suggests that submission victories—as a more overarching variable for grappling—may be better suited to test the grappler hypothesis, and might further suggest that the grappler hypothesis may be better supported by ground‐based grappling variables.

While Study 1 found significant but small effects of upper limb length on overall fighting, knockout, and submission success, associations between morphology and fighting performance using real‐world contest data are typically small (Caton, Hannan, and Dixson 2022a; Richardson 2021; Třebický et al. 2013; Třebický et al. 2015; Zilioli et al. 2015). This may be particularly the case in skilled populations where fighting skill is theorized to play a more dominant role in determining outcomes than morphological traits (Caton, Pearson, and Dixson 2022b; Kirk 2018). Previous research has indeed attended to statistical significance over effect size in these contexts, as the small effect sizes observed in elite populations align with expectations given the complex interplay of factors influencing performance (Caton, Pearson, and Dixson 2022b). While less skilled populations may provide opportunities to detect larger effects, data availability remains a challenge, as the necessary anthropometric and performance metrics are primarily collected by elite fighting organizations that can afford to gather and maintain such extensive data. Even if associations remain small in less skilled groups, this may still align with evolutionary expectations: while the advantages of longer upper limbs may be mitigated by an opponents' ability to adapt through skill or speed, even minor benefits that consistently improved survival or reproductive success could have driven the evolution of upper limb length, influenced sexual dimorphism, and shaped co‐evolving psychological traits.

Following on from Study 1, Study 2 hypothesized male‐biased sexual dimorphism in upper limb length, after accounting for allometry. If selection favored longer upper limbs in humans for the purposes of intrasexual combat, and intrasexual combat was more common among men than women (and therefore created greater selection pressures for upper limb length in men than women), then we should expect humans to exhibit unique sexual dimorphism in upper limb length. Studies 2a–2d provided evidence for this unique sexual dimorphism.

### 5.1 Limitations and Future Directions

An additional consideration for the evolution of upper limb length is that upper limb length may have evolved to increase throwing, and therefore hunting, power. This is not mutually exclusive to the argument that upper limb length evolved to increase fighting success; indeed, there are complementary hypotheses in the human evolutionary literature which posit that human morphological features evolved to increase fighting ability (Sell et al. 2012; Sell et al. 2016; Sell et al. 2017) and hunting ability (Apicella 2014; Eisenbruch et al. 2024; Smith et al. 2017). For upper limb length, Raschka et al. (2015) found that greater upper limb length is not associated javelin throwing power, but that professional javelin throwers have shorter upper limbs than amateur javelin throwers. However, world class javelin throwers are tall (Sharma and Mukhopadhyay 2022), tall javelin throwers outperform shorter javelin throwers (Ul Haq et al. 2020), and height is positively correlated with span (Table 1). Nonetheless, research has questioned whether javelin throwers are the best model for hunter‐gatherer throwing (e.g., Roach et al. 2013).

The contest advantages of upper limb length extend to a range of sports (Ackland et al. 2009). Debanne and Laffaye (2011) found that span predicted ball throwing velocity in male handball players and Karadenizli (2016) found that span predicted ball throwing velocity in female handball players. Baseball pitchers categorized as high velocity throwers exhibit longer upper limbs than those categorized as low velocity throwers (Matsuo et al. 2001). Goranovic et al. (2023) found that that upper limb length was the largest positive predictor of ball throwing velocity in professional handball players. Singh and Shukla (2024) found positive associations between upper limb length and the number of successful free throws, three‐point throws, and maximum accurate throw distance in basketball. In a review of kinanthropometric measurements and sports performance, Quraishi et al. (2022) note that greater upper limb length contributes to performance in sports requiring single long shots, including pole vaulters, discus throwers and javelin. Future research should examine whether upper limb length (controlling for allometry) contributes to throwing ability, with application to hunting. This research could draw on existing methodologies from human evolutionary research, such as examining morphological measurements (in this case, upper limb length) and hunting ability in the Hadza (see Apicella 2014).

Human contest competition research has relied on UFC data to test evolutionary hypotheses, as UFC data is regarded a good proxy of human contest competition because fights are limited by a few regulations only (e.g., strikes to genitals and eyes are forbidden) and are highly aggressive (Dixson et al. 2017; Schild and Zettler 2021). However, there are the following limitations, many of which are discussed in recent research (Caton and Dixson 2022a; Caton, Hannan, and Dixson 2022a; Caton, Pearson, and Dixson 2022b; Caton et al. 2024). First, and namely for the present work, the UFC (and all other fighting performance organizations) do not measure biacromial width directly and this measure must be derived from photograph‐based landmarking procedures; however, we note these photograph‐derived measurements exhibit positive correlations with all other anthropometric measurements (for further discussion, see Supporting Information). Second, the UFC's weight categories may limit the effect of morphological features on fighting performance. Third, the UFC represents the best combatants in the world and effects could be substantially reduced in highly‐skilled fighting environments, given that psychophysical systems responsible for fighting skill are argued to have evolved in response to the contest advantages associated with larger morphology (Caton, Hannan, and Dixson 2022a; Caton, Pearson, and Dixson 2022b; Caton et al. 2024). Fourth, UFC combatants may exhibit extreme muscle mass and training patterns, providing a less realistic parallel than other athletic populations and reducing the true advantage of static morphological features. Following recommendations to observe the effects of morphology in less‐skilled fighting populations (Caton and Dixson 2022a; Caton, Pearson, and Dixson 2022b), recent research has observed larger effects for morphology in less‐skilled environments (Caton et al. 2024). Future research is encouraged to observe whether upper limb length exhibits increased effect sizes in environments where morphological features are hypothesized to exhibit greater advantages.

Fifth, mixed‐martial‐arts differs from ancestral combat in that it does not incorporate weapons and the question of whether upper limb length confers an advantage in weapon‐based fights remains open for exploration. Most broadly, Caton et al. (2024) discuss that the emergence of weapons underscores the evolutionary importance of morphological features in human evolution. Given that longer upper limbs may allow for greater work and peak power (Lockie et al. 2018; but also see Fett et al. 2020; Lätt et al. 2010; Rozi et al. 2018) and exhibit advantages across a range of sports (Ackland et al. 2009), we might expect that the contest advantages of upper limb length extend to weapon use (e.g., greater work and peak power when using knives or swords). Furthermore, if the emergence of combat‐based weapons indeed evolved in response to the contest advantages associated with increased morphology, then we may also expect that certain weapons mimic the advantages associated with longer upper limbs (e.g., polearm, glaive, halberd, pike, poleaxe); that is, the invention of certain weapons may have been inspired by morphological features. Future research is encouraged to explore these exciting new avenues, which would underscore the importance of upper limb length in contest competition but also demonstrate the influence of male morphology on the history of weapons.

While the present work has focused on human contest competition, research also suggests that selection has favored reduced aggression and increased prosocial behaviors in humans (Sánchez‐Villagra and Van Schaik 2019). It is important to note that the evolution of cooperation and competition are interdependent (Ito and Doebeli 2019) and war drives prosocial behavior (Gneezy and Fessler 2012; also see parochial altruism, which pairs ingroup favoritism with outgroup hostility; Choi and Bowles 2007). Carrier and Morgan (2014) note reduced robusticity in morphological features, such as craniofacial robusticity, may have been due to improved weapon technology. This increase in weapon technology would have provided salient visual cues that serve as reminders of mortality, and mortality salience increases prosocial behavior (Jonas et al. 2002). Ultimately, the resulting decreased importance of physical formidability does not undermine, but rather may underscore, the evolutionary importance of hand‐to‐hand combat (for a more thorough discussion that is beyond the scope of the present work, see Caton et al. 2024).

While the present work has provided a range of evidence for a potential new human anatomical characteristic shaped by sexual selection, there may also be alternative explanations for sexual dimorphism in upper limb length (e.g., growth patterns, sex‐specific ecological strategies). Growth patterns and sexual selection are not mutually exclusive but can be complementary and interdependent (Dixson 2009; Hodges‐Simeon et al. 2016; Hodges‐Simeon et al. 2021; Setchell and Lee 2004). Growth patterns can influence the development of traits that are then subject to sexual selection (e.g., if a male's delayed puberty results in greater height or muscle mass, these traits can be favored by sexual selection) and sexual selection can shape growth patterns (e.g., if taller males are preferred, natural selection might favor growth patterns that result in increased height) (Hodges‐Simeon et al. 2021; also see Dixson 2009; see Stulp and Barrett 2016, for an overview of the evolution of human height which considers sexual dimorphism as evidence of sexual selection, with the exception of sex‐specific ecological strategies, which we note as a possible alternative explanation to sexual dimorphism in upper limb length).

It is important to note that a single study cannot determine whether a human anatomical characteristic is shaped by sexual selection; rather, whether a feature has been shaped by sexual selection broadly, or is sexually dimorphic specifically, should be subject to ongoing research (e.g., facial width‐to‐height ratio [fWHR] research; see Hodges‐Simeon et al. 2021). Hodges‐Simeon et al. (2021) discussed the requirements for secondary sexual characteristics and provided an overview of the various paradigms used to examine whether fWHR was shaped by sexual selection. Given the large and ongoing nature of determining whether a characteristic has been shaped by sexual selection, this presents a large opportunity for future research (for an overview of research on fWHR, which have implications for research on upper limb length, see Hodges‐Simeon et al. 2021).

Given the potential for upper limb length to represent a human anatomical characteristic uniquely shaped by sexual selection (e.g., facial width‐to‐height ratio: Carré and McCormick 2008; waist‐to‐hip ratio: Singh 1993), the current studies point toward several important future directions for biological and psychological research. Such characteristics in men predict numerous social and psychological outcomes, including greater wealth (Wong et al. 2011), political attitudes (Sell et al. 2017), career progress (Mueller and Mazur 1996), body satisfaction (Frederick et al. 2007), romantic desirability (Sidari et al. 2021), aggression and adolescent bullying (Sell et al. 2016), and greater political success (Todorov et al. 2005). Future research could profit from testing currently unknown relationships between *Homo sapiens* upper limb length and this constellation of psychological outcomes, and, in doing so, extend the findings of the current studies beyond contest competition research and into the many domains of the biological and psychological sciences.

## Ethics Statement

The authors have nothing to report.

## Conflicts of Interest

The authors declare no conflicts of interest.

## Supporting information


**Data S1** Supporting Information.

## Data Availability

All data needed to evaluate the conclusions in the paper are provided on the Open Science Framework (https://osf.io/v3qpn/).

## Endnotes

^1^
Darwin (1872) specifically argued that *Homo sapiens* have evolved specialized psychological systems—rage—which amplify those physiological (e.g., muscular exertion, heightened blood oxygenation) and anatomical (e.g., upper appendages) features already implicated in fighting success. Thus, Darwin's (1872) argument was first predicated on the premise that human anatomical structures—the upper limbs—have evolved to increase damage infliction in violent combat, but that the rage‐based emotional system *amplifies* the effectiveness of anatomical structures (see Darwin 1872, *Chapter X*: *Hatred and Anger*).

^2^
Richardson (2021) only examined the association between span and one fighting‐related variable: win percentage (i.e., total wins divided by total fights). The entire results section is eight sentences long, and there are no additional analyses in a supplemental material section.

^3^
Likewise, Dixson et al. (2017) only found an association between span and win percentage. Dixson et al. (2017) also examined—but, importantly for the originality the present study, did not find—associations between span, knockout victories, and decision victories. Further important to the originality of the present work, Dixson et al. (2017) did not examine span in relation to submission victories, and no other fighting‐variables were examined in the study. It should also be noted that the covariation for important variables was not controlled for in Dixson et al. (2017) (e.g., body size, leg length, age, experience [i.e., fighter's debut date]). Dixson et al. (2017) also used a fight‐specific approach, which has been questioned by more recent research and recent unsuccessful replication attempts in contest competition research (for a more thorough discussion on fight‐specific versus overall fighter data, see Caton, Hannan, and Dixson 2022a).

^4^
Further important to the present work, no study has examined upper limb *length* in real‐world contest data because real‐world fight data only includes information on span; no study has proposed methodology for disentangling the covariation between upper limb length and biacromial width, which is conflated within the span variable. The present work proposed a methodology for disentangling this covariation, allowing future researchers to examine the unique variation associated with upper limb length and biacromial width in real‐world contest data. For example, the present work found that the biacromial width of UFC fighters exhibited expected positive associations with weight, height, leg length, span, and exhibited male‐biased sexual dimorphism (see Table 1). There was also a positive bivariate association between biacromial width and knockout success, providing support for the long‐standing hypothesis that biacromial width is a *cue* to success in real‐world contest competition (e.g., Puts 2010; Sell et al. 2012).

## References

[ajhb70010-bib-0001] Ackland, T. R. , B. Elliott , and J. Bloomfield . 2009. Applied Anatomy and Biomechanics in Sport. Human Kinetics.

[ajhb70010-bib-0002] Adams, D. C. , and E. Otárola‐Castillo . 2013. “Geomorph: An R Package for the Collection and Analysis of Geometric Morphometric Shape Data.” Methods in Ecology and Evolution 4, no. 4: 393–399. 10.1111/2041-210X.12035.

[ajhb70010-bib-0003] Aggarwal, A. N. , D. Gupta , L. M. Ezekiel , and S. K. Jindal . 2000. “Statistical Estimation of Height From Arm Span in North Indian Subjects.” Indian Journal of Physiology and Pharmacology 44: 329–334. 10941622

[ajhb70010-bib-0004] Andersson, M. 1994. Sexual Selection. Princeton University Press.

[ajhb70010-bib-0005] Apicella, C. L. 2014. “Upper‐Body Strength Predicts Hunting Reputation and Reproductive Success in Hadza Hunter–Gatherers.” Evolution and Human Behavior 35, no. 6: 508–518. 10.1016/j.evolhumbehav.2014.07.001.

[ajhb70010-bib-0006] Arnott, G. , and R. W. Elwood . 2009. “Assessment of Fighting Ability in Animal Contests.” Animal Behaviour 77, no. 5: 991–1004. 10.1016/j.anbehav.2009.02.010.

[ajhb70010-bib-0007] Aung, T. , S. Goetz , J. Adams , et al. 2021. “Low Fundamental and Formant Frequencies Predict Fighting Ability Among Male Mixed Martial Arts Fighters.” Scientific Reports 11: 1–10. 10.1038/s41598-020-79408-6. 33441596 PMC7806622

[ajhb70010-bib-0008] Behringer, V. , J. M. Stevens , T. L. Kivell , J. Neufuss , C. Boesch , and G. Hohmann . 2016. “Within Arm's Reach: Measuring Forearm Length to Assess Growth Patterns in Captive Bonobos and Chimpanzees.” American Journal of Physical Anthropology 161, no. 1: 37–43. 10.1002/ajpa.23004. 27143225

[ajhb70010-bib-0009] Bertamini, M. , and K. M. Bennett . 2009. “The Effect of Leg Length on Perceived Attractiveness of Simplified Stimuli.” Journal of Social, Evolutionary, and Cultural Psychology 3: 233–250. 10.1037/h0099320.

[ajhb70010-bib-0010] Bjelica, D. , S. Popović , M. Kezunović , J. Petković , G. Jurak , and P. Grasgruber . 2012. “Body Height and Its Estimation Utilising Arm Span Measurements in Montenegrin Adults.” Anthropological Notebooks 18: 69–83.

[ajhb70010-bib-0012] Bookstein, F. L. 1997. Morphometric Tools for Landmark Data. Cambridge University Press.

[ajhb70010-bib-0013] Bramble, D. M. , and D. E. Lieberman . 2004. “Endurance Running and the Evolution of Homo.” Nature 432, no. 7015: 345–352. 10.1038/nature03052. 15549097

[ajhb70010-bib-0014] Briffa, M. , and S. M. Lane . 2017. “The Role of Skill in Animal Contests: A Neglected Component of Fighting Ability.” Proceedings of the Royal Society B: Biological Sciences 284, no. 1863: 20171596. 10.1098/rspb.2017.1596. PMC5627213 28954913

[ajhb70010-bib-0015] Carré, J. M. , and C. M. McCormick . 2008. “In Your Face: Facial Metrics Predict Aggressive Behaviour in the Laboratory and in Varsity and Professional Hockey Players.” Proceedings of the Royal Society B: Biological Sciences 275, no. 1651: 2651–2656. 10.1098/rspb.2008.0873. PMC2570531 18713717

[ajhb70010-bib-0016] Carrier, D. R. 2011. “The Advantage of Standing up to Fight and the Evolution of Habitual Bipedalism in Hominins.” PLoS One 6, no. 5: e19630. 10.1371/journal.pone.0019630. 21611167 PMC3097185

[ajhb70010-bib-0017] Carrier, D. R. , and M. H. Morgan . 2014. “Protective Buttressing of the Hominin Face.” Biological Reviews 90, no. 1: 330–346. 10.1111/brv.12112. 24909544

[ajhb70010-bib-0018] Caton, N. R. , L. M. Brown , A. A. Z. Zhao , and B. J. Dixson . 2024. “Human Male Body Size Predicts Increased Knockout Power, Which Is Accurately Tracked by Conspecific Judgments of Male Dominance.” Human Nature 35: 114–133. 10.1007/s12110-024-09473-7. 38878141 PMC11317448

[ajhb70010-bib-0019] Caton, N. R. , and B. J. Dixson . 2022a. “Human Third‐Party Observers Accurately Track Fighting Skill and Vigour Along Their Unique Paths to Victory.” Scientific Reports 12, no. 1: 14841. 10.1038/s41598-022-19044-4. 36050502 PMC9437099

[ajhb70010-bib-0020] Caton, N. R. , and B. J. Dixson . 2022b. “Beyond Facial Width‐To‐Height Ratios: Bizygomatic Width Is Highly Sexually Dimorphic When Adjusting for Allometry.” Biology Letters 18, no. 10: 20220211. 10.1098/rsbl.2022.0211.

[ajhb70010-bib-0021] Caton, N. R. , J. Hannan , and B. J. Dixson . 2022a. “Facial Width‐To‐Height Ratio Predicts Fighting Success: A Direct Replication and Extension of Zilioli et al. (2014).” Aggressive Behavior 48, no. 5: 449–465. 10.1002/ab.22027. 35262921 PMC9544882

[ajhb70010-bib-0022] Caton, N. R. , S. G. Pearson , and B. J. Dixson . 2022b. “Is Facial Structure an Honest Cue to Real‐World Dominance and Fighting Ability in Men? A Pre‐Registered Direct Replication of Třebický et al. (2013).” Evolution and Human Behavior 43, no. 4: 314–324. 10.1016/j.evolhumbehav.2022.04.002.

[ajhb70010-bib-0023] Caton, N. R. , S. G. Pearson , and B. J. W. Dixson . 2023. “A Re‐Analysis That Replicated a Replication: Rejoinder to Třebický, Havlíček, and Kleisner (2022).” Evolution and Human Behavior 44, no. 2: 161–167. 10.1016/j.evolhumbehav.2023.01.008.

[ajhb70010-bib-0024] Choi, J. K. , and S. Bowles . 2007. “The Coevolution of Parochial Altruism and War.” Science 318, no. 5850: 636–640. 10.1126/science.1144237. 17962562

[ajhb70010-bib-0025] Cieri, R. L. , S. E. Churchill , R. G. Franciscus , J. Tan , and B. Hare . 2014. “Craniofacial Feminization, Social Tolerance, and the Origins of Behavioral Modernity.” Current Anthropology 55, no. 4: 419–443. 10.1086/677209.

[ajhb70010-bib-0026] Darwin, C. 1872. The Expression of the Emotions in Man and Animals. *J*. *Murray* .

[ajhb70010-bib-0027] Debanne, T. , and G. Laffaye . 2011. “Predicting the Throwing Velocity of the Ball in Handball With Anthropometric Variables and Isotonic Tests.” Journal of Sports Sciences 29, no. 7: 705–713. 10.1080/02640414.2011.552112. 21400345

[ajhb70010-bib-0028] Dixson, A. F. 2009. Sexual Selection and the Origins of Human Mating Systems. Oxford University Press.

[ajhb70010-bib-0029] Dixson, B. J. , J. M. Sherlock , W. K. Cornwell , and M. M. Kasumovic . 2017. “Contest Competition and Men's Facial Hair: Beards May Not Provide Advantages in Combat.” Evolution and Human Behavior 39: 147–153. 10.1016/j.evolhumbehav.2017.11.004.

[ajhb70010-bib-0030] Eisenbruch, A. B. , K. M. Smith , C. I. Workman , C. von Rueden , and C. L. Apicella . 2024. “US Adults Accurately Assess Hadza and Tsimane Men's Hunting Ability From a Single Face Photograph.” Evolution and Human Behavior 45, no. 4: 106598. 10.1016/j.evolhumbehav.2024.106598.

[ajhb70010-bib-0031] Emlen, D. J. 2008. “The Evolution of Animal Weapons.” Annual Review of Ecology, Evolution, and Systematics 39: 387–413. 10.1146/annurev.ecolsys.39.110707.173502.

[ajhb70010-bib-0032] Erawan, B. , S. T. Paramitha , D. Mulyana , and M. G. Ramadhan . 2020. “The Digitalization of Wrestling Basic Techniques for Learning.” In 4th International Conference on Sport Science, Health, and Physical Education (ICSSHPE 2019), 178–182. Atlantis Press. 10.2991/ahsr.k.200214.048.

[ajhb70010-bib-0033] Fenton, T. W. , J. L. DeJong , and R. C. Haut . 2003. “Punched With a Fist: The Etiology of a Fatal Depressed Cranial Fracture.” Journal of Forensic Sciences 48, no. 2: 277–281. 12664983

[ajhb70010-bib-0034] Fett, J. , A. Ulbricht , and A. Ferrauti . 2020. “Impact of Physical Performance and Anthropometric Characteristics on Serve Velocity in Elite Junior Tennis Players.” Journal of Strength & Conditioning Research 34, no. 1: 192–202. 10.1519/JSC.0000000000002641. 29912079

[ajhb70010-bib-0035] Finni, T. , H. de Brito Fontana , and H. Maas . 2023. “Force Transmission and Interactions Between Synergistic Muscles.” Journal of Biomechanics 152: 111575. 10.1016/j.jbiomech.2023.111575. 37120913

[ajhb70010-bib-0036] Floyd, B. 2017. “How Much Impact Do Gains in Height Have on Shoulder Breadths Within Taiwanese Families?” American Journal of Human Biology 29: e22991. 10.1002/ajhb.22991. 28266086

[ajhb70010-bib-0037] Flynn, A. , M. Halsey , and M. Lee . 2016. “Emblematic Violence and Aetiological Cul‐de‐Sacs: On the Discourse of ‘One‐Punch’(Non) Fatalities.” British Journal of Criminology 56, no. 1: 179–195. 10.1093/bjc/azv039.

[ajhb70010-bib-0038] Frederick, D. A. , G. M. Buchanan , L. Sadehgi‐Azar , et al. 2007. “Desiring the Muscular Ideal: Men's Body Satisfaction in the United States, Ukraine, and Ghana.” Psychology of Men & Masculinity 8, no. 2: 103–117. 10.1037/1524-9220.8.2.103.

[ajhb70010-bib-0039] Gneezy, A. , and D. M. Fessler . 2012. “Conflict, Sticks and Carrots: War Increases Prosocial Punishments and Rewards.” Proceedings of the Royal Society B: Biological Sciences 279, no. 1727: 219–223. 10.1098/rspb.2011.0805. PMC3223676 21653590

[ajhb70010-bib-0040] Goranovic, K. , J. Petkovic , M. Joksimovic , S. Karisik , and N. Eler . 2023. “The Influence of Morphological Characteristics on the Ball Throwing Velocity in the Professional Handball Players.” International Journal of Morphology 41, no. 6: 1881–1886. 10.4067/S0717-95022023000601881.

[ajhb70010-bib-0041] Hånell, A. , and E. Rostami . 2020. “How Can a Punch Knock You out?” Frontiers in Neurology 11: 1–6. 10.3389/fneur.2020.570566. 33193016 PMC7649325

[ajhb70010-bib-0042] Hayes, A. F. 2013. Introduction to Mediation, Moderation, and Conditional Process Analysis. A Regression‐Based Approach. Guilford Press.

[ajhb70010-bib-0043] Herzog, W. 2017. “Skeletal Muscle Mechanics: Questions, Problems and Possible Solutions.” Journal of Neuroengineering and Rehabilitation 14: 1–17. 10.1186/s12984-018-0351-5. 28915834 PMC5603017

[ajhb70010-bib-0044] Hill, A. K. , D. H. Bailey , and D. A. Puts . 2017. “Gorillas in Our Midst? Human Sexual Dimorphism and Contest Competition in Men.” In On Human Nature, 235–249. Academic Press.

[ajhb70010-bib-0045] Hodges‐Simeon, C. R. , G. Albert , G. B. Richardson , et al. 2021. “Was Facial Width‐To‐Height Ratio Subject to Sexual Selection Pressures? A Life Course Approach.” PLoS One 16, no. 3: e0240284. 10.1371/journal.pone.0240284. 33711068 PMC7954343

[ajhb70010-bib-0046] Hodges‐Simeon, C. R. , K. N. Hanson Sobraske , T. Samore , M. Gurven , and S. J. Gaulin . 2016. “Facial Width‐To‐Height Ratio (fWHR) is Not Associated With Adolescent Testosterone Levels.” PLoS One 11, no. 4: e0153083. 10.1371/journal.pone.0153083. 27078636 PMC4831733

[ajhb70010-bib-0047] Ito, K. , and M. Doebeli . 2019. “The Joint Evolution of Cooperation and Competition.” Journal of Theoretical Biology 480: 1–12. 10.1016/j.jtbi.2019.07.010. 31323234

[ajhb70010-bib-0048] James, L. P. , S. Robertson , G. G. Haff , E. M. Beckman , and V. G. Kelly . 2017. “Identifying the Performance Characteristics of a Winning Outcome in Elite Mixed Martial Arts Competition.” Journal of Science and Medicine in Sport 20, no. 3: 296–301. 10.1016/j.jsams.2016.08.001. 27569006

[ajhb70010-bib-0049] Jonas, E. , J. Schimel , J. Greenberg , and T. Pyszczynski . 2002. “The Scrooge Effect: Evidence That Mortality Salience Increases Prosocial Attitudes and Behavior.” Personality and Social Psychology Bulletin 28, no. 10: 1342–1353. 10.1177/014616702236834.

[ajhb70010-bib-0050] Karadenizli, Z. I. 2016. “The Relationships Between Ball Throwing Velocity and Physical‐Psychomotor Features for Talent Identification in Physical Education.” Universal Journal of Educational Research 4, no. 11: 2509–2515. 10.13189/ujer.2016.041103.

[ajhb70010-bib-0051] Katić, R. , S. Blažević , S. Krstulović , and R. Mulić . 2005. “Morphological Structures of Elite Karateka and Their Impact on Technical and Fighting Efficiency.” Collegium Antropologicum 29, no. 1: 79–84. 16117303

[ajhb70010-bib-0052] Kirk, C. 2018. “Does Anthropometry Influence Technical Factors in Competitive Mixed Martial Arts?” Human Movement Science 19, no. 2: 46–59. 10.5114/hm.2018.74059.

[ajhb70010-bib-0053] Lane, S. M. , and M. Briffa . 2020. “Perceived and Actual Fighting Ability: Determinants of Success by Decision, Knockout or Submission in Human Combat Sports.” Biology Letters 16, no. 10: 20200443. 10.1098/rsbl.2020.0443. 33108983 PMC7655483

[ajhb70010-bib-0054] Lätt, E. , J. Jürimäe , J. Mäestu , et al. 2010. “Physiological, Biomechanical and Anthropometrical Predictors of Sprint Swimming Performance in Adolescent Swimmers.” Journal of Sports Science and Medicine 9, no. 3: 398–404. 24149633 PMC3761703

[ajhb70010-bib-0055] Lee, Y. C. , C. H. Lee , and C. H. Chen . 2020. “Replication Data for Body Anthropometric Measurements of Singaporean Elderly Population (V2).” Harvard Dataverse. 10.7910/DVN/JSA627.

[ajhb70010-bib-0056] Leit, R. A. , H. G. Pope Jr. , and J. J. Gray . 2001. “Cultural Expectations of Muscularity in Men: The Evolution of Playgirl Centerfolds.” International Journal of Eating Disorders 29, no. 1: 90–93. 10.1002/1098-108x(200101)29:1<90::aid-eat15>3.0.co;2-f. 11135340

[ajhb70010-bib-0057] Lieber, R. L. , and S. R. Ward . 2011. “Skeletal Muscle Design to Meet Functional Demands.” Philosophical Transactions of the Royal Society, B: Biological Sciences 366, no. 1570: 1466–1476. 10.1098/rstb.2010.0316. PMC3130443 21502118

[ajhb70010-bib-0059] Lo, M. S. , L. L. Lin , W. J. Yao , and M. C. Ma . 2011. “Training and Detraining Effects of the Resistance vs. Endurance Program on Body Composition, Body Size, and Physical Performance in Young Men.” Journal of Strength & Conditioning Research 25, no. 8: 2246–2254. 10.1519/JSC.0b013e3181e8a4be. 21747300

[ajhb70010-bib-0060] Lockie, R. G. , M. R. Moreno , A. J. Orjalo , et al. 2018. “Relationships Between Height, Arm Length, and Leg Length on the Mechanics of the Conventional and High‐Handle Hexagonal Bar Deadlift.” Journal of Strength & Conditioning Research 32, no. 11: 3011–3019. 10.1519/JSC.0000000000002256. 29045317

[ajhb70010-bib-0061] Maas, H. , and T. G. Sandercock . 2010. “Force Transmission Between Synergistic Skeletal Muscles Through Connective Tissue Linkages.” BioMed Research International 2010, no. 1: 575672. 10.1155/2010/575672. PMC2853902 20396618

[ajhb70010-bib-0062] Matsuo, T. , R. F. Escamilla , G. S. Fleisig , S. W. Barrentine , and J. R. Andrews . 2001. “Comparison of Kinematic and Temporal Parameters Between Different Pitch Velocity Groups.” Journal of Applied Biomechanics 17, no. 1: 1–13. 10.1123/jab.17.1.1.

[ajhb70010-bib-0063] McCullough, E. L. , C. W. Miller , and D. J. Emlen . 2016. “Why Sexually Selected Weapons Are Not Ornaments.” Trends in Ecology & Evolution 31: 742–751. 10.1016/j.tree.2016.07.004. 27475833

[ajhb70010-bib-0064] McCullough, E. L. , B. W. Tobalske , and D. J. Emlen . 2014. “Structural Adaptations to Diverse Fighting Styles in Sexually Selected Weapons.” Proceedings of the National Academy of Sciences 111: 14484–14488. 10.1073/pnas.1409585111. PMC4209975 25201949

[ajhb70010-bib-0065] Mirzaei, B. , D. G. Curby , I. Barbas , and N. Lotfi . 2013. “Differences in Some Physical Fitness and Anthropometric Measures Between Greco‐Roman and Freestyle Wrestlers.” International Journal of Wrestling Science 3, no. 1: 94–102. 10.1080/21615667.2013.10878973.

[ajhb70010-bib-0066] Mitchell, C. O. , and D. A. Lipschitz . 1982. “Arm Length Measurement as an Alternative to Height in Nutritional Assessment of the Elderly.” Journal of Parenteral and Enteral Nutrition 6, no. 3: 226–229. 10.1177/0148607182006003226. 7202060

[ajhb70010-bib-0067] Moreno, E. 2011. “The Society of Our “out of Africa” Ancestors (I) the Migrant Warriors That Colonized the World.” Communicative & Integrative Biology 4, no. 2: 163–170. 10.4161/cib.4.2.14320. 21655430 PMC3104569

[ajhb70010-bib-0068] Morgan, M. H. , and D. R. Carrier . 2013. “Protective Buttressing of the Human Fist and the Evolution of Hominin Hands.” Journal of Experimental Biology 216, no. 2: 236–244. 10.1242/jeb.075713. 23255192

[ajhb70010-bib-0069] Morris, J. S. , C. B. Cunningham , and D. R. Carrier . 2019. “Sexual Dimorphism in Postcranial Skeletal Shape Suggests Male‐Biased Specialization for Physical Competition in Anthropoid Primates.” Journal of Morphology 280, no. 5: 731–738. 10.1002/jmor.20980. 30892726

[ajhb70010-bib-0070] Morris, J. S. , J. Link , J. C. Martin , and D. R. Carrier . 2020. “Sexual Dimorphism in Human Arm Power and Force: Implications for Sexual Selection on Fighting Ability.” Journal of Experimental Biology 223, no. 2: jeb212365. 10.1242/jeb.212365. 31862852

[ajhb70010-bib-0071] Moshkdanian, G. , S. Mahaki Zadeh , F. Moghani Ghoroghi , T. Mokhtari , and G. Hassanzadeh . 2014. “Estimation of Stature From the Anthropometric Measurement of Lower Limb in Iranian Adults.” Anatomical Sciences Journal 11, no. 3: 149–154.

[ajhb70010-bib-0072] Mueller, U. , and A. Mazur . 1996. “Facial Dominance of West Point Cadets as a Predictor of Later Military Rank.” Social Forces 74, no. 3: 823–850. 10.1093/sf/74.3.823.

[ajhb70010-bib-0073] Nakagawa, S. , F. Kar , R. E. O'Dea , J. L. Pick , and M. Lagisz . 2017. “Divide and Conquer? Size Adjustment With Allometry and Intermediate Outcomes.” BMC Biology 15: 1–6. 10.1186/s12915-017-0448-5. 29121927 PMC5679152

[ajhb70010-bib-0074] Nor, F. M. , N. Abdullah , A. M. Mustapa , L. Q. Wen , N. A. Faisal , and D. A. A. A. Nazari . 2013. “Estimation of Stature by Using Lower Limb Dimensions in the Malaysian Population.” Journal of Forensic and Legal Medicine 20, no. 8: 947–952. 10.1016/j.jflm.2013.09.006. 24237796

[ajhb70010-bib-0075] Oishi, M. , N. Ogihara , H. Endo , N. Ichihara , and M. Asari . 2009. “Dimensions of Forelimb Muscles in Orangutans and Chimpanzees.” Journal of Anatomy 215, no. 4: 373–382. 10.1111/j.1469-7580.2009.01125.x. 19619166 PMC2766055

[ajhb70010-bib-0076] Paquette, S. 2009. Anthropometric Survey (ANSUR) II Pilot Study: Methods and Summary Statistics. Anthrotch, US Army Natick Soldier Research, Development and Engineering Center.

[ajhb70010-bib-0077] Parke, W. C. 2020. Biophysics: A Student's Guide to the Physics of the Life Sciences and Medicine. Springer Nature.

[ajhb70010-bib-0079] Puts, D. , D. Carrier , and A. R. Rogers . 2023. “Contest Competition for Mates and the Evolution of Human Males.” In The Oxford Handbook of Human Mating, 317–377. Oxford University Press.

[ajhb70010-bib-0080] Puts, D. A. 2010. “Beauty and the Beast: Mechanisms of Sexual Selection in Humans.” Evolution and Human Behavior 31, no. 3: 157–175. 10.1016/j.evolhumbehav.2010.02.005.

[ajhb70010-bib-0081] Puts, D. A. , D. H. Bailey , and P. L. Reno . 2015. “Contest Competition in Men.” In The Handbook of Evolutionary Psychology, 1–18. Wiley and Sons. 10.1002/9781119125563.evpsych113.

[ajhb70010-bib-0082] Quanjer, P. H. , A. Capderou , M. M. Mazicioglu , et al. 2014. “All‐Age Relationship Between Arm Span and Height in Different Ethnic Groups.” European Respiratory Journal 44: 905–912. 10.1183/09031936.00054014. 25063245

[ajhb70010-bib-0083] Quraishi, S. , A. Chahal , V. Esht , et al. 2022. “Kinanthropometric Measurements: A Better Understanding From an Athlete's Perspective.” Saudi Journal of Sports Medicine 22, no. 3: 89–93. 10.4103/sjsm.sjsm_5_21.

[ajhb70010-bib-0084] Raschka, C. , K. Vöth , and K. Kuczera . 2015. “Sports Anthropological and Somatotypical Comparison Between Young Male Shotputters and Javelin Throwers of Different Performance Classes and Recreational Athletes.” Papers on Anthropology 24, no. 1: 129–141. 10.12697/poa.2015.24.1.11.

[ajhb70010-bib-0085] Richardson, T. 2021. “Is Arm Length a Sexually Selected Trait in Humans? Evidence From Mixed Martial Arts.” Evolutionary Behavioral Sciences 15, no. 2: 175. 10.1037/ebs0000219.supp.

[ajhb70010-bib-0116] Richardson, T. , and R. T. Gilman . 2019. “Left‐Handedness Is Associated With Greater Fighting Success in Humans.” Scientific Reports 9, no. 1: 15402. 10.1038/s41598-019-51975-3. 31659217 PMC6817864

[ajhb70010-bib-0086] Roach, N. T. , M. Venkadesan , M. J. Rainbow , and D. E. Lieberman . 2013. “Elastic Energy Storage in the Shoulder and the Evolution of High‐Speed Throwing in Homo.” Nature 498, no. 7455: 483–486. 10.1038/nature12267. 23803849 PMC3785139

[ajhb70010-bib-0087] Rohlf, J. F. 2018. tpsDig2 (Version 2.31) [Computer Software]. Department of Ecology and Evolution, State University of New York at Stony Brook.

[ajhb70010-bib-0088] Rozi, G. , V. Thanopoulos , N. Geladas , E. Soultanaki , and M. Dopsaj . 2018. “Anthropometric Characteristics and Physiological Responses of High Level Swimmers and Performance in 100 m Freestyle Swimming.” Movement & Sport Sciences—Science & Motricité 101: 3–7. 10.1051/sm/2018007.

[ajhb70010-bib-0089] Ruff, C. 1987. “Sexual Dimorphism in Human Lower Limb Bone Structure: Relationship to Subsistence Strategy and Sexual Division of Labor.” Journal of Human Evolution 16, no. 5: 391–416. 10.1016/0047-2484(87)90069-8.

[ajhb70010-bib-0090] Sánchez‐Villagra, M. R. , and C. P. Van Schaik . 2019. “Evaluating the Self‐Domestication Hypothesis of Human Evolution.” Evolutionary Anthropology: Issues, News, and Reviews 28, no. 3: 133–143. 10.1002/evan.21777. 30938920

[ajhb70010-bib-0091] Schild, C. , and I. Zettler . 2021. “Linking Voice Pitch to Fighting Success in Male Amateur Mixed Martial Arts Athletes and Boxers.” Evolutionary Human Sciences 3: e46. 10.1017/ehs.2021.45. 37588524 PMC10427264

[ajhb70010-bib-0092] Schoonaert, K. , K. D'Août , and P. Aerts . 2007. “Morphometrics and Inertial Properties in the Body Segments of Chimpanzees (*Pan troglodytes*).” Journal of Anatomy 210, no. 5: 518–531. 10.1111/j.1469-7580.2007.00720.x. 17451529 PMC2375742

[ajhb70010-bib-0093] Sell, A. , M. Eisner , and D. Ribeaud . 2016. “Bargaining Power and Adolescent Aggression: The Role of Fighting Ability, Coalitional Strength, and Mate Value.” Evolution and Human Behavior 37, no. 2: 105–116. 10.1016/j.evolhumbehav.2015.09.003.

[ajhb70010-bib-0094] Sell, A. , L. S. Hone , and N. Pound . 2012. “The Importance of Physical Strength to Human Males.” Human Nature 23, no. 1: 30–44. 10.1007/s12110-012-9131-2. 22477166

[ajhb70010-bib-0095] Sell, A. , D. Sznycer , L. Cosmides , et al. 2017. “Physically Strong Men Are More Militant: A Test Across Four Countries.” Evolution and Human Behavior 38, no. 3: 334–340. 10.1016/j.evolhumbehav.2016.11.002.

[ajhb70010-bib-0096] Setchell, J. M. , and P. C. Lee . 2004. “Development and Sexual Selection in Primates.” In Sexual Selection in Primates: New and Comparative Perspectives, edited by P. M. Kappeler and C. P. van Schaik , 175–195. Cambridge University Press. 10.1017/CBO9780511542459.012.

[ajhb70010-bib-0097] Sharma, B. , and K. Mukhopadhyay . 2022. “Analysis of Selected Physical Parameters of Olympic Gold Medalist Shot Putters.” Russian Journal of Physical Education and Sport 17, no. 4: 17–25. 10.14526/2070-4798-2022-17-4-18-26.

[ajhb70010-bib-0098] Sidari, M. J. , A. J. Lee , S. C. Murphy , J. M. Sherlock , B. J. Dixson , and B. P. Zietsch . 2021. “Preferences for Sexually Dimorphic Body Characteristics Revealed in a Large Sample of Speed Daters.” Social Psychological and Personality Science 12, no. 2: 225–236. 10.1177/1948550619882925.

[ajhb70010-bib-0099] Singh, A. , and A. Shukla . 2024. “Co‐Relation Between Arm Length and Basketball Throw.” International Journal of Contemporary Research in Multidisciplinary 3, no. 3: 75–77. 10.5281/zenodo.11581490.

[ajhb70010-bib-0100] Singh, D. 1993. “Adaptive Significance of Female Physical Attractiveness: Role of Waist‐To‐Hip Ratio.” Journal of Personality and Social Psychology 65, no. 2: 293–307. 10.1037/0022-3514.65.2.293. 8366421

[ajhb70010-bib-0101] Smith, K. M. , Y. M. Olkhov , D. A. Puts , and C. L. Apicella . 2017. “Hadza Men With Lower Voice Pitch Have a Better Hunting Reputation.” Evolutionary Psychology 15, no. 4: 1474704917740466. 10.1177/1474704917740466. 29179581 PMC10481060

[ajhb70010-bib-0102] Stulp, G. , and L. Barrett . 2016. “Evolutionary Perspectives on Human Height Variation.” Biological Reviews 91, no. 1: 206–234. 10.1111/brv.12165. 25530478

[ajhb70010-bib-0103] Todorov, A. , A. N. Mandisodza , A. Goren , and C. C. Hall . 2005. “Inferences of Competence From Faces Predict Election Outcomes.” Science 308, no. 5728: 1623–1626. 10.1126/science.1110589. 15947187

[ajhb70010-bib-0104] Třebický, V. , J. Fialová , K. Kleisner , S. C. Roberts , A. C. Little , and J. Havlíček . 2015. “Further Evidence for Links Between Facial Width‐To‐Height Ratio and Fighting Success: Commentary on Zilioli et al. (2014).” Aggressive Behavior 41: 331–334. 10.1002/ab.21559. 25236530

[ajhb70010-bib-0106] Třebický, V. , J. Havlíček , S. C. Roberts , A. C. Little , and K. Kleisner . 2013. “Perceived Aggressiveness Predicts Fighting Performance in Mixed‐Martial‐Arts Fighters.” Psychological Science 24, no. 9: 1664–1672. 10.1177/0956797613477117. 23818656

[ajhb70010-bib-0107] Ul Haq, M. Z. , T. Arif , and M. A. Nawaz . 2020. “Angular Kinematics and Physical Fitness Analysis of Tall Height and Short Height Javelin Throwers—A Case Study of the Islamia University of Bahawalpur, Pakistan.” Journal of Business and Social Review in Emerging Economies 6, no. 2: 829–833. 10.26710/jbsee.v6i2.1255.

[ajhb70010-bib-0108] Wilson, R. S. , G. K. David , S. C. Murphy , et al. 2017. “Skill Not Athleticism Predicts Individual Variation in Match Performance of Soccer Players.” Proceedings of the Royal Society B: Biological Sciences 284, no. 1868: 20170953. 10.1098/rspb.2017.0953. PMC5740267 29187623

[ajhb70010-bib-0109] Wong, E. M. , M. E. Ormiston , and M. P. Haselhuhn . 2011. “A Face Only an Investor Could Love: CEOs' Facial Structure Predicts Their Firms' Financial Performance.” Psychological Science 22, no. 12: 1478–1483. 10.1177/0956797611418838. 22042727

[ajhb70010-bib-0110] Wrangham, R. W. 2019. “Hypotheses for the Evolution of Reduced Reactive Aggression in the Context of Human Self‐Domestication.” Frontiers in Psychology 10: 1914. 10.3389/fpsyg.2019.01914. 31481917 PMC6710405

[ajhb70010-bib-0111] Zatsiorsky, V. M. , and B. I. Prilutsky . 2012. Biomechanics of Skeletal Muscles. Human Kinetics.

[ajhb70010-bib-0112] Zihlman, A. L. 1997. “Natural History of Apes: Life History Features in Females and Males.” In Evolving Female: A Life History Perspective, 86–103. Princeton University Press. 10.1515/9781400822065.86.

[ajhb70010-bib-0113] Zihlman, A. L. , and R. K. McFarland . 2000. “Body Mass in Lowland Gorillas: A Quantitative Analysis.” American Journal of Physical Anthropology 113, no. 1: 61–78. 10.1002/1096-8644(200009)113:1<61::AID-AJPA6>3.0.CO;2-H. 10954620

[ajhb70010-bib-0114] Zilioli, S. , A. N. Sell , M. Stirrat , J. Jagore , W. Vickerman , and N. V. Watson . 2015. “Face of a Fighter: Bizygomatic Width as a Cue of Formidability.” Aggressive Behavior 41, no. 4: 322–330. 10.1002/ab.21544. 24910133

[ajhb70010-bib-0115] Živičnjak, M. , N. S. Narančić , L. Szirovicza , D. Franke , J. Hrenović , and V. Bišof . 2003. “Gender‐Specific Growth Patterns for Stature, Sitting Height and Limbs Length in Croatian Children and Youth (3 to 18 Years of Age).” Collegium Antropologicum 27, no. 1: 321–334. 12974162

