# Supplementary material for: Intrasexual Selection for Upper Limb Length in Homo sapiens
Source: Am J Hum Biol. 2025 Feb 19;37(2):e70010. doi: 10.1002/ajhb.70010 (PMC11837467; doi:10.1002/ajhb.70010)
Supplement: Supplementary file 1 — Data S1 Supporting Information. [file AJHB-37-e70010-s001.docx]

**Supplemental Material**

**Method**

Demographic data were collected for fighter’s age (*M*_years_ = 30.62; *SD*_years_ = 4.53), professional debut date (15^th^ May, 1998, to 9^th^ November, 2019), span (*M*_centimetres_ = 182.37; *SD*_centimetres_ = 11.42), lower limb length (*M*_centimetres_ = 102.00; *SD*_centimetres_ = 7.58), weight (*M*_kgs_ = 74.29; *SD*_kgs_ = 16.65), height (*M*_centimetres_ = 177.65; *SD*_centimetres_ = 9.34), and fighter’s sex (113 females, 602 males). The UFC introduced lower limb length (leg reach) measurements over a decade ago to their publicly available morphological measurements (Bohn, 2014). The UFC measures lower limb length from the hipbone to the heel (Bohn, 2014). While there is no publicly available information on which areas within the UFC measure the lower limb length of the fighters, or the background or training of the observers, we note that a hipbone to the heel measurement is a relatively straightforward measurement. This measurement may originate from the UFC Performance Institute (<https://ufc-pi.webflow.io/>), a $12 million performance institute in Nevada, United States, that is the UFC's centre for innovation, research and training and reports (Pugmire, 2017). The UFC Performance Institute Journal, available on their website, reports publicly available descriptive statistics across a range of anthropometric and physiological measurements of UFC fighters, but we are unaware of any detailed descriptions of the observers who measure the lower limb length measurement.

We further collected data on total wins (*M* = 14.54; *SD* = 7.08), losses (*M* = 4.52; *SD* = 3.51), and draws (*M* = .13; *SD* = .39), which we summed to compute a “total fights” variable (*M* = 19.20; *SD* = 9.88). In line with prior research (Caton, Pearson, & Dixson, 2022; Zilioli et al., 2015), fighting success was then calculated as total wins divided by total fights (*M* = .77; *SD* = .12). Detailed information on the nature of wins (i.e., by knockout or technical knockout [KO/TKO]: *M* = 5.90; *SD* = 4.24; submission: *M* = 4.39; *SD* = 4.17; decision: *M* = 4.24; *SD* = 3.19) was collected from espn.com. Detailed information on the nature of losses (i.e., by knockout or technical knockout: *M* = 1.29; *SD* = 1.56; submission: *M* = 1.05; *SD* = 1.47; decision: *M* = 2.19; *SD* = 2.17) were also drawn from espn.com. Finally, data on fighter’s lifetime (1) striking accuracy (*M* = 44.78; *SD* = 9.35), (2) strike defense (*M* = 56.11; *SD* = 9.42), (3) takedown defense (*M* = 66.34; *SD* = 19.81), and (4) grappling accuracy (*M* = 42.18; *SD* = 22.92) were collected from ufc.com, as this data was not available on espn.com.

UFC facial photographs have been advantageous for previous research because fighters are similarly postured and positioned at approximately similar distances from the camera (Caton, Hannan, & Dixson, 2022; Caton, Pearson, & Dixson, 2022; Zilioli et al., 2015). After collecting fighters’ statistics and facial photographs (standardised: equally sized at 350 x 254 pixels), four independent research assistants performed facial landmarking procedures on the entire collection of 715 faces (i.e., approximately 180 faces per research assistant) in the tpsDig2 software (version 2.31; Rohlf, 2018a). Landmarking is a widely-used technique in the biological sciences that is used to describe the length, width, and/or shape of morphological traits, where individual landmarks are used to capture anatomically similar positions in different stimuli (Adams & Otárola‐Castillo, 2013; Bookstein, 1997; Caton, Hannan, & Dixson 2022; Caton, Pearson, & Dixson, 2022). Landmarking procedures have been previously validated for the human psychological sciences, where they have been applied to research on facial (but not bodily) structure and fighting ability (Caton, Pearson & Dixson, 2022; Zilioli et al., 2015).

To measure biacromial width, research assistants landmarked the intersection of the lower attachment of the upper trapezius muscle and upper attachment of the deltoid (shoulder) muscles (Figure S1). This anatomical location served as a more anatomically specific description of the superior acromion for research assistants, as opposed to asking research assistants to simply landmark the superior acromion. There were no landmarking errors for these two landmarks as indicated using the *plotOutliers* function in the *Geomorph* package in R. We then computed the distance between these landmarks to create two key variables that had not been investigated in previous contest competition research: biacromial width and upper limb length. Controlling for biacromial width allows us to examine the unique variance associated with upper limb length.

**Figure S1**

*Biacromial Width Landmarking in TpsDig2*.


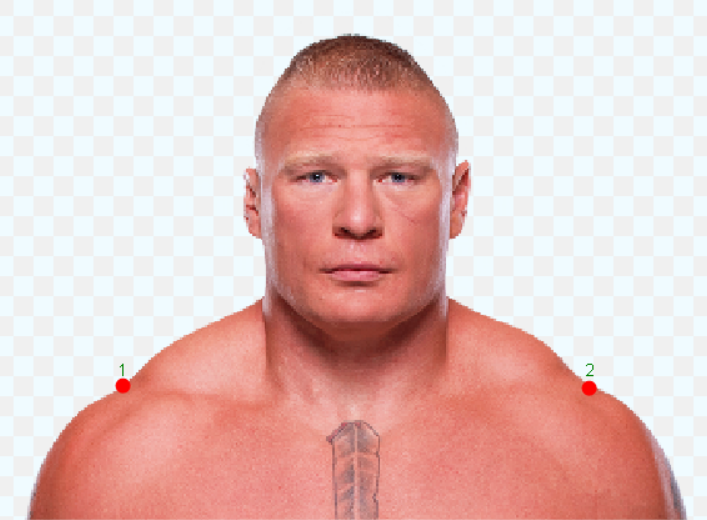


*Note.* Official UFC image for Brock Lesnar, landmarked in tpsDig2 for biacromial width.

Regarding the validation of morphological measurements from UFC photographs, while we are unaware of any documented process for UFC photographs, deriving morphological measurements from UFC facial photographs has been widely employed in human contest competition research (e.g., Zilioli et al., 2015; Třebický et al., 2013; Třebický et al., 2015; Caton, Hannan, & Dixson, 2022; Caton, Pearson, & Dixson, 2022). As noted above, we standardised images in the sense that the images are equally sized at 350 x 254 pixels but the photographs themselves are approximately standardised (e.g., in that there is approximately similar lighting, background, neutral expression, and distance from the camera). We assume that the same camera and setup is *not* used for all individuals examined but infer from the photographs themselves (and from the convergent associations discussed below) that this is approximate. While these photographs are not perfectly standardised, human contest competition research has noted the advantages of using UFC photographs for their similarities between images (e.g., Caton, Pearson, & Dixson, 2022; Little et al., 2015). For the present work, convergent validity (i.e., the degree to which two measures that theoretically should exhibit associations do indeed exhibit associations; in this case, particularly other morphological measurements) may be the most straightforward form of validation for these measurements. Indeed, biacromial width derived from the UFC photographs exhibited convergent validity with other morphological measures such that significant positive associations were observed with all other morphological measurements, including span, span-to-height, leg length, height and weight (Table 1). Biacromial width derived from the UFC photographs also exhibited positive correlations with knockout successes as well as sex, with biacromial width exhibiting male-biased sexual dimorphism as expected (Table 1). While it would be ideal to use an actual morphological measurement for biacromial width, only a photograph-derived measurement of biacromial width can be extracted for real-world contest data given that biacromial width is not collected by mixed-martial-arts or other fighting performance organisations. This means that this is the only method by which to directly test these human contest competition hypotheses, but we nonetheless acknowledge this presents a limitation of the present work.

***The Distance Hypothesis***

A mediation analysis was conducted to test the distance hypothesis (H_2_): that upper limb length is associated with greater fighting success through increased striking accuracy, striking defense, and/or grappling defense. We controlled for biacromial width, leg length, height, weight, age, debut date, total fights, and sex. In line with the recommendations of Hayes (2013), the significance of the mediated (i.e., indirect) effects were examined using bootstrapping procedures. Bootstrapping uses the original sample as the population from which random samples with replacement are used to provide the best estimate of the true indirect effect. Employing the Hayes (2013) SPSS PROCESS macro (model 4; v.4.1), 10000 bootstrap samples were created to estimate bias-corrected standard errors and 95% percentile confidence intervals for the indirect effect. The indirect effect is considered significant at *p* < .05 if zero is not included in its 95% confidence interval.

Findings are depicted in Figure S2. Results of the bias-corrected bootstrapped analyses found that upper limb length did not have a significant indirect effect on fighting success via striking accuracy (*ab* path = .00003, bootstrap *SE* = .0001, 95% [-.0003, .0003]), striking defense (*ab* path = -.0001, bootstrap *SE* = .0002, 95% [-.0005, .0002]), or grappling defense (*ab* path = -.0001, bootstrap *SE* = .0001, 95% [-.0004, .0002]). The presence of zero within these confidence interval ranges does not support the distance hypothesis that striking accuracy, striking defense, or grappling defense would significantly mediate the relationship between upper limb length and fighting success. The full model accounted for 43.5% of the variance in fighting success.

**Figure S2**

*The Distance Hypothesis*

Strike Accuracy

.002 (*p* = .004)

.02 (*p* = .80)

Upper Limb Length

Fighting Success

*c* = .002 (*p* = .14)

*c’* = .002 (*p* = .09)

.002 (*p* = .01)

-.07 (*p* = .37)

Striking Defense

-.13 (*p* = .48)

.001 (*p* = .004)

Grappling Defense

*Note.* Unstandardised regression coefficients for the relationship between upper limb length and fighting success as mediated by striking accuracy and strike and takedown defense. Biacromial width, leg length, height, weight, age, debut date, total fights, and sex were included as covariates but are not included in the figure.

***The Grappler Hypothesis***

A mediation analysis was conducted to test the grappler hypothesis (H_2_): that upper limb length is associated with greater fighting success through increased grappling accuracy. We again controlled for biacromial width, leg length, height, weight, age, debut date, total fights, and sex. Findings are depicted in Figure S3. Results of the bias-corrected bootstrapped analyses found that upper limb length did not have a significant indirect effect on fighting success via grappling accuracy (*ab* path = .00003, bootstrap *SE* = .0001, 95% CI [-.0002, .0003]. The presence of zero within the confidence interval range does not support the grappler hypothesis that grappling accuracy would significantly mediate the relationship between upper limb length and fighting success. The full model accounted for 36.3% of the variance in fighting success.

**Figure S3**

*The Grappler Hypothesis*

Grappling Accuracy

*b* =.0001 (*p* = .72)

*a* = .33 (*p* = .12)

Upper Limb Length

Fighting Success

*c* = .002 (*p* = .07)

*c’* = .002 (*p* = .07)

*Note.* Unstandardised regression coefficients for the relationship between upper limb length and fighting success as mediated by grappling accuracy. Biacromial width, leg length, height, weight, age, debut date, total fights, and sex were included as covariates but are not included in the figure.

**Study 2**

**Study 2d**

Below is the SPSS syntax for the grouping of subjects’ birth locations (countries,

states) into their respective world region (i.e., Africa, Europe, Asia, Oceania, and North, Central, and South America):

***Central America***

recode SubjectsBirthLocation (“Antigua and Barbuda”=1)(“Costa Rica”=1)(“Barbados”=1)

(“Belize”=1)(“Cuba”=1)(“El Salvador”=1)(“El Salvador”=1)(“Grenada”=1)(“Bermuda”=1)

(“Bahamas”=1)(“Haiti”=1)(“Honduras”=1)(“Jamaica”=1)(“Puerto Rico”=1)

(“Dominica”=1)(“Dominican Republic”=1)(“Grenada”=1)(“Guadalupe”=1)(“Guatemala”=1)

(“Trinidad and Tobago”=1)(“Nicaragua”=1)(“British Virgin Islands”=1)(“Mexico”=1)

(“Panama”=1)(“Saint Lucia”=1)(“US Virgin Islands”=1)

(else=sysmis) into CentralAm.

***North America***

recode SubjectsBirthLocation (“Canada”=1)(“Alabama”=1)(“Alaska”=1)(“Arizona”=1)

(“Arkansas”=1)

(“California”=1)(“Colorado”=1)(“Connecticut”=1)(“Delaware”=1)(“District of

Columbia"=1)

("Florida"=1)("Georgia"=1)("Florida"=1)

("Idaho"=1)("Illinois"=1)("Indiana"=1)("Iowa"=1)("Kansas"=1)("Kentucky"=1)

("Louisiana"=1)

("Maine"=1)("Maryland"=1)("Massachusetts"=1)("Michigan"=1)("Minnesota"=1)

("Mississippi"=1)

("Missouri"=1)("Montana"=1)("Nebraska"=1)("Nevada"=1)("New Hampshire"=1)

("New Jersey"=1)("New Mexico"=1)("New York"=1)("North Carolina"=1)("North

Dakota"=1)

("Ohio"=1)("Oklahoma"=1)("Oregon"=1)("Pennsylvania"=1)("Rhode Island"=1)

("South Carolina"=1)("South Dakota"=1)("Tennessee"=1)("Texas"=1)

("United States"=1)("Utah"=1)("Vermont"=1)("Virginia"=1)("Washington"=1)("West

Virginia"=1)

("Wisconsin"=1)("Wyoming"=1)(else=sysmis) into NorthAm.

***Africa***

recode SubjectsBirthLocation ("Cape Verde"=1)("Cameroon"=1)("Nigeria"=1)

("Sierra Leone"=1)("South Africa"=1)("Sudan"=1)("Morocco"=1)("Egypt"=1)("Ghana"=1)

("Ethiopia"=1)("Ivory Coast"=1)("Kenya"=1)("Liberia"=1)("Senegal"=1)("Togo"=1)

("Zambia"=1)

(else=sysmis) into Africa.

***Europe***

recode SubjectsBirthLocation ("Belgium"=1)("France"=1)("Germany"=1)("Iceland"=1)

("Italy"=1)("Portugal"=1)("Netherlands"=1)("Poland"=1)("Romania"=1)("Bosnia and

Herzegovina"=1)

("Russia"=1)("Ukraine"=1)("United Kingdom"=1)("Bulgaria"=1)("Belarus"=1)

("Denmark"=1)("Serbia"=1)

(else=sysmis) into Europe.

***Asia***

recode SubjectsBirthLocation ("Azerbaijan"=1)("Bangladesh"=1)("Burma"=1)

("Cambodia"=1)("China"=1)("India"=1)("Iran"=1)("Iraq"=1)("Israel"=1)

("Japan"=1)("Korea"=1)("Laos"=1)("Lebanon"=1)("Turkey"=1)("Nepal"=1)

("Philippines"=1)

("Singapore"=1)("South Korea"=1)("Sri Lanka"=1)("Syria"=1)("Taiwan"=1)("Thailand"=1)

("Vietnam"=1)

(else=sysmis) into Asia.

***South America***

recode SubjectsBirthLocation ("Argentina"=1)("Bolivia"=1)("Brazil"=1)

("Chile"=1)("Colombia"=1)("Ecuador"=1)("French Guiana"=1)("Guyana"=1)("Paraguay"=1)

("Peru"=1)("South America"=1)("Venezuela"=1)

(else=sysmis) into SouthAmerica.

***Oceania***

recode SubjectsBirthLocation ("American Samoa"=1)("Fiji"=1)("Guam"=1)("Hawaii"=1)

("Micronesia"=1)("New Zealand"=1)("Northern Mariana Islan"=1)

("Palau"=1)

(else=sysmis) into Pacific

**Analyses Without Controlling for Biacromial Width**

All analyses were then conducted without the biacromial width variable controlled for. Other than this one distinction, the process for all analyses remained exactly the same as the previous analyses that included biacromial width. As per previous discussions, we refer to arm span without controlling for biacromial width as arm span; we only refer to models with arm span controlling for biacromial width as upper limb length. Therefore, all below analyses in this section (“*Analyses without Biacromial Width*”) refer to arm span.

To summarise the below, most analyses remained the same without including biacromial width. First, this does not mean that biacromial width is not an important variable because, without controlling for biacromial width and observing the relative effect, researchers cannot ensure that their upper limb length variables reflect upper limb length itself. The below results further ensure that arm span, as used in previous studies (e.g., Dixson et al., 2017; Richardson, 2021), is likely to reflect upper limb length, which is beneficial for the broader literature. Second, biacromial width exhibited positive bivariate associations with other morphological features (e.g., weight, height, leg length, span) and knockout success (see Table 1) indicating its benefit as a control variable due to overlapping variance. Third, previous studies (e.g., Dixson et al., 2017; Richardson, 2021) only found associations between span and overall fighting success, whereas our study reports novel associations between upper limb length and span and knockout and submission success and further examined novel associations between upper limb length and span and striking accuracy and defense and grappling accuracy and defense.

Fourth, despite the above, it may still be queried whether controlling for biacromial width was necessary—per the below, controlling for biacromial width demonstrably proved important in the examination of upper limb length on fighting success via submission success. It was demonstrated earlier that upper limb *length* (i.e., controlling for biacromial width) predicted fighting success via submission success. The below analyses, when biacromial width is removed, demonstrates that there is no significant *indirect* effect of arm span on fighting success via submission success. There was, however, direct associations between arm span and submission success, submission success and fighting success, and arm span and fighting success. However, these direct associations were not substantial enough to result in a significant *indirect* effect when not controlling for biacromial width. While biacromial width does not significantly predict either submission success (*p* = .23) or fighting success (*p* = .73) when included in the model, the fact that removing biacromial width changes this indirect effect from significant to non-significant does reflect some overlapping variance between biacromial width on the associations between arm span and submission and fighting success. In any case, even when biacromial width is removed, there are nonetheless direct associations between arm span and submission success, submission success and fighting success, and arm span and fighting success—but cleaner results are found in submission success analyses when controlling for biacromial width, emphasising the need to control for biacromial width.

First, we tested whether arm span increases fighting success through knockout success. A mediation analysis (SPSS PROCESS macro; model 4; v.4.1; 10,000 bootstrap samples; Hayes, 2013) was run for the relation between arm span and fighting success as mediated by knockout success, controlling for weight, height, lower limb length, age, debut date, total fights, and sex. When measuring knockout victories, we also controlled for submission and decision victories, as these are statistically related (but theoretically distinct) avenues to victory (Caton & Dixson, 2022a; Lane & Briffa, 2020).

Results supported the hypothesis that arm span was associated with fighting success (Figure S4). Results of the bias-corrected bootstrapped analyses further supported that arm span exhibited a significant indirect effect on fighting success via knockout success (*ab* path = .002, bootstrap *SE* = .0009, 95% CI [.0002, .004]).

**Figure S4**

*Arm Span Increases Fighting Success through Knockout Success*

Knockout Success

*a* = .04 (*p* = .01)

*b* = .05 (*p* < .001)

Arm Span

Fighting Success

*c* = .002 (*p* = .02)

*c’* = .0003 (*p* = .59)

*Note.* Unstandardised regression coefficients (with *p*-values in brackets) for the relationship between arm span and fighting success as mediated by knockout success. Lower limb length, height, weight, age, debut date, total fights, sex, and wins by submission and decision were included as covariates but are not included in the figure. Indirect (a*b), direct (c’) and total effects (c) can be found in the figure.

A mediation analysis (SPSS PROCESS macro; model 4; v.4.1; 10,000 bootstrap samples; Hayes, 2013) was run for the relation between arm span and fighting success as mediated by submission success, controlling for weight, height, lower limb length, age, debut date, total fights, and sex. When measuring submission victories, we also controlled for knockout and decision victories. As discussed above, results of the bias-corrected bootstrapped analyses found that arm span did not exhibit a significant indirect effect on fighting success via submission success (*ab* path = .002, bootstrap *SE* = .0009, 95% CI [-.00006, .003]). As elaborated on above, Figure S5 reports significant direct associations between arm span and submission success, submission success and fighting success, and arm span and fighting success.

**Figure S5**

*Arm Span Increases Fighting Success via Submission Success*

Submission Success

*b* =.05 (p < .001)

*a* = .04 (*p* = .02)

Arm Span

Fighting Success

*c* = .002 (*p* = .04)

*c’* = .0003 (p = .59)

*Note.* Unstandardised regression coefficients (with *p*-values in brackets) for the relationship between arm span and fighting success as mediated by submission success. Lower limb length, height, weight, age, debut date, total fights, sex, and wins by KO/TKO and decision were included as covariates but are not included in the figure. Indirect (a*b), direct (c’) and total effects (c) can be found in the figure.

A mediation analysis was also conducted to test whether arm span is associated with greater fighting success through increased grappling accuracy. We again controlled for leg length, height, weight, age, debut date, total fights, and sex. Findings are depicted in Figure S6. Results of the bias-corrected bootstrapped analyses found that arm span did not have a significant indirect effect on fighting success via grappling accuracy (*ab* path = .00002, bootstrap *SE* = .0001, 95% CI [-.0002, .0003]. The presence of zero within the confidence interval range does not support that grappling accuracy would significantly mediate the relationship between arm span and fighting success. The full model accounted for 36.3% of the variance in fighting success.

**Figure S6**

*The Grappler Hypothesis*

Grappling Accuracy

*a* = .26 (*p* = .21)

*b* = .0001 (*p* = .73)

Arm Span

Fighting Success

*c* = .002 (*p* = .06)

*c’* = .002 (*p* = .06)

*Note.* Unstandardised regression coefficients for the relationship between arm span and fighting success as mediated by grappling accuracy. Leg length, height, weight, age, debut date, total fights, and sex were included as covariates but are not included in the figure.

***The Distance Hypothesis***

A mediation analysis was conducted to test whether arm span is associated with greater fighting success through increased striking accuracy, striking defense, and/or grappling defense. We controlled for leg length, height, weight, age, debut date, total fights, and sex. Findings are depicted in Figure S7. Results of the bias-corrected bootstrapped analyses found that arm span did not have a significant indirect effect on fighting success via striking accuracy (*ab* path = .00002, bootstrap *SE* = .0001, 95% [-.0003, .0003]), striking defense (*ab* path = -.0001, bootstrap *SE* = .0001, 95% [-.0005, .0002]), or grappling defense (*ab* path = -.0001, bootstrap *SE* = .0001, 95% [-.0004, .0002]). The presence of zero within these confidence interval ranges does not support that striking accuracy, striking defense, or grappling defense would significantly mediate the relationship between arm span and fighting success. The full model accounted for 43.5% of the variance in fighting success.

**Figure S7**

*The Distance Hypothesis*

Strike Accuracy

.002 (*p* = .004)

.02 (*p* = .83)

Arm Span

Fighting Success

*c* = .001 (*p* = .14)

*c’* = .002 (*p* = .08)

.002 (*p* = .01)

-.08 (*p* = .27)

Striking Defense

-.16 (*p* = .37)

.001 (*p* = .004)

Grappling Defense

*Note.* Unstandardised regression coefficients for the relationship between arm span and fighting success as mediated by striking accuracy and strike and takedown defense. Leg length, height, weight, age, debut date, total fights, and sex were included as covariates but are not included in the figure.

**Biacromial Width Analyses**

We also examined all analyses previously reported for upper limb length, but with biacromial width as the focal variable of interest (controlling for arm span). Given that biacromial width was measured using landmarking techniques applied to facial and body photographs (e.g., per Figure S1, described earlier), unstandardised coefficients for this variable are interpreted such that a one-unit increase in biacromial width represents a one-pixel increase. To summarise the below, all associations between biacromial width and those variables related to contest competition were non-significant. This does not mean that biacromial width was not an important variable for inclusion in the models focussed on upper limb length, as biacromial width exhibited positive bivariate associations with other morphological features (e.g., weight, height, leg length, span) and knockout success (see Table 1) and its removal affected the significance of the indirect effect (but not the individual direct associations) of span on fighting success via submission success, as discussed earlier. Thus, the below represents that biacromial width is not associated with variables implicated in contest competition when controlling for other demographic and morphological variables, which suggests that biacromial width is dependent on other variables for its initial bivariate association with knockout success.

A mediation analysis (SPSS PROCESS macro; model 4; v.4.1; 10,000 bootstrap samples; Hayes, 2013) was run for the relation between biacromial width and fighting success as mediated by knockout success, controlling for span, weight, height, lower limb length, age, debut date, total fights, and sex. When measuring knockout victories, we also controlled for submission and decision victories, as these are statistically related (but theoretically distinct) avenues to victory (Caton & Dixson, 2022a; Lane & Briffa, 2020).

Results did not support that biacromial width was associated with fighting success (Figure S8). Results of the bias-corrected bootstrapped analyses showed that biacromial width did not exhibit a significant indirect effect on fighting success via knockout success (*ab* path = -.00005, bootstrap *SE* = .0002, 95% CI [-.0005, .0004]).

**Figure S8**

*Biacromial Width Does Not Increase Fighting Success through Knockout Success*

Knockout Success

*a* = -.001 (*p* = .84)

*b* =.05 (p < .001)

Biacromial Width

Fighting Success

*c* = .0001 (*p* = .69)

*c’* = .0002 (p = .35)

*Note.* Unstandardised regression coefficients (with *p*-values in brackets) for the relationship between biacromial width and fighting success as mediated by knockout success. Span, lower limb length, height, weight, age, debut date, total fights, sex, and wins by submission and decision were included as covariates but are not included in the figure. Indirect (a*b), direct (c’) and total effects (c) can be found in the figure.

A mediation analysis (SPSS PROCESS macro; model 4; v.4.1; 10,000 bootstrap samples; Hayes, 2013) was run for the relation between biacromial width and fighting success as mediated by submission success, controlling for span, weight, height, lower limb length, age, debut date, total fights, and sex. In line with the above analysis, when measuring submission victories, we also controlled for knockout and decision victories. Findings are depicted in Figure S9. Results of the bias-corrected bootstrapped analyses showed that biacromial width did not exhibit a significant indirect effect on fighting success via submission success (*ab* path = -.0003, bootstrap *SE* = .0002, 95% CI [-.0006, .0002]).

**Figure S9**

*Biacromial Width Does Not Increase Fighting Success via Submission Success*

Submission Success

*a* = -.006 (*p* = .23)

*b* =.05 (p < .001)

Biacromial Width

Fighting Success

*c* = -.0001 (*p* = .73)

*c’* = .0002 (*p* = .35)

*Note.* Unstandardised regression coefficients (with *p*-values in brackets) for the relationship between biacromial width and fighting success as mediated by submission success. Span, lower limb length, height, weight, age, debut date, total fights, sex, and wins by KO/TKO and decision were included as covariates but are not included in the figure. Indirect (a*b), direct (c’) and total effects (c) can be found in the figure.

A mediation analysis was conducted to test whether biacromial width is associated with greater fighting success through increased grappling accuracy. We again controlled for span, leg length, height, weight, age, debut date, total fights, and sex. Findings are depicted in Figure S10. Results of the bias-corrected bootstrapped analyses found that biacromial width did not have a significant indirect effect on fighting success via grappling accuracy (*ab* path = -.00001, bootstrap *SE* = .00004, 95% CI [-.0001, .0001]. The presence of zero within the confidence interval range does not support that grappling accuracy would significantly mediate the relationship between biacromial width and fighting success. The full model accounted for 36.3% of the variance in fighting success.

**Figure S10**

*Biacromial Width Does Not Increase Fighting Success via Grappling Accuracy*

Grappling Accuracy

*b* =.0001 (*p* = .72)

*a* = -.12 (*p* = .06)

Biacromial Width

Fighting Success

C = .00005 (*p* = .87)

C’ = .0001 (*p* = .85)

*Note.* Unstandardised regression coefficients for the relationship between biacromial width and fighting success as mediated by grappling accuracy. Span, leg length, height, weight, age, debut date, total fights, and sex were included as covariates but are not included in the figure.

A mediation analysis was conducted to test whether biacromial width is associated with greater fighting success through increased striking accuracy, striking defense, and/or grappling defense. We controlled for span, leg length, height, weight, age, debut date, total fights, and sex. Findings are depicted in Figure S11. Results of the bias-corrected bootstrapped analyses found that biacromial width did not have a significant indirect effect on fighting success via striking accuracy (*ab* path = .00001, bootstrap *SE* = .00004, 95% [-.0001, .0001]), striking defense (*ab* path = .00003, bootstrap *SE* = .00004, 95% [-.0001, .0001]), or grappling defense (*ab* path = -.00004, bootstrap *SE* = .00004, 95% [-.0001, .00004]). The presence of zero within these confidence interval ranges does not support that striking accuracy, striking defense, or grappling defense would significantly mediate the relationship between biacromial width and fighting success. The full model accounted for 43.5% of the variance in fighting success.

**Figure S11**

*The Distance Hypothesis for Biacromial Width*

Strike Accuracy

.002 (*p* = .004)

-.005 (*p* = .83)

Biacromial Width

Fighting Success

*c* = .00004 (*p* = .88)

*c’* = .00003 (*p* = .90)

.002 (*p* = .01)

-.02 (*p* = .31)

Striking Defense

-.05 (*p* = .34)

.001 (*p* = .004)

Grappling Defense

*Note.* Unstandardised regression coefficients for the relationship between biacromial width and fighting success as mediated by striking accuracy and strike and takedown defense. Span, leg length, height, weight, age, debut date, total fights, and sex were included as covariates but are not included in the figure.

**Allometric and Absolute Sexual Dimorphism in Upper Limb Length**

Table S1 reports the allometric and absolute dimorphism across each of the populations examined in Study 2. This table notes the control variables used for each of the analyses. These analyses differ from those analyses reported in the present work’s main text solely in that the main text analyses further controlled for demographic variables. The below allometric analyses controlled for the morphological variables reported in the main text whereas the absolute analyses do not control for these morphological variables. Only Study 2a’s (controlling for biacromial width) absolute analyses include a control variable – biacromial width – to ensure that this row reflects upper limb length. However, Study 2a’s (without controlling for biacromial row) absolute analyses do not include biacromial width as a control variable.

**Table S1. Allometric and Absolute Sexual Dimorphism in Upper Limb Length**

|  | Allometric upper limb length dimorphism | | Absolute upper limb length dimorphism | |
| --- | --- | --- | --- | --- |
|  | *B* | *p* | *B* | *p* |
| Study 2a (controlling for biacromial width) | **2.83** | **< .001** | **16.36** | **< .001** |
| Study 2a (without controlling for biacromial width) | **3.25** | **< .001** | **17.30** | **< .001** |
| Study 2b | **.48** | **< .001** | 2.32 | .59 |
| Study 2c | **1.86** | **.002** | **4.57** | **< .001** |
| Study 2d (entire sample) | **.79** | **< .001** | **6.98** | **< .001** |

*Note.* For clarity, B represents unstandardised B coefficients (i.e., in this case, the degree to which males exceed females in centimetres for upper limb length). Study 2a allometric analyses reported in this table control for height, weight and lower limb length. For Study 2a allometric analyses, the first allometric analyses row further control for biacromial width, whereas the second row allometric analyses controls for the aforementioned variables but not biacromial width. For Study 2a’s absolute analyses, the first row’s absolute analyses control for biacromial width to ensure that variable reflects arm length; for the second row’s absolute analyses, these absolute analyses contain no control variables. For Study 2b-2d, no absolute analyses include any control variables. Study 2b allometric analyses reported in this table control for stature and lower limb length. Study 2c allometric analyses reported in this table control for stature and lower limb length. Study 2d allometric analyses reported in this table control for stature, lower limb length and weight.

In addition to the unstandardised coefficients reported in Table S1, which document the difference in upper limb length between males and females, Table S2 reports the descriptive statistics (mean, median, standard deviation, and minimum and maximum scores) for span in study 2a and upper limb length across studies 2b-2d.

**Table S2. Summary Statistics for Upper Limb Length**

|  | Overall | | | | Male | | | | Female | | | |
| --- | --- | --- | --- | --- | --- | --- | --- | --- | --- | --- | --- | --- |
|  | *M* | *Mdn* | *SD* | *Min - Max* | *M* | *Mdn* | *SD* | *Min - Max* | *M* | *Mdn* | *SD* | *Min - Max* |
| Study 2a (span) | 182.37 | 182.88 | 11.42 | 153.67 to 214.63 | 185.04 | 185.42 | 10.01 | 157.48 to 214.63 | 167.75 | 167.64 | 6.57 | 153.67 to 185.42 |
| Study 2b | 64.41 | 66.15 | 11.87 | 43.10 to 81.50 | 65.57 | 65.85 | 13.00 | 43.10 to 81.50 | 63.25 | 66.50 | 10.93 | 43.20 to 73.90 |
| Study 2c | 75.66 | 76.10 | 4.08 | 65.80 to 84.70 | 77.94 | 78.60 | 3.33 | 69.50 to 84.70 | 73.37 | 73.40 | 3.46 | 65.80 to 81.30 |
| Study 2d (entire sample) | 82.11 | 82.50 | 5.08 | 61.30 to 99.00 | 84.39 | 84.40 | 3.92 | 70.90 to 99.00 | 77.42 | 77.20 | 3.81 | 61.30 to 91.20 |

*Note.* Study 2a uses arm span (as previously mentioned, arm length as its own individual variable is not available in this dataset) and summary statistics require that individual variables are used. Following on from previous analyses and discussions, Study 2b-2d use arm length (centimetres). *M* = mean, *Mdn* = median, *SD* = standard deviation, and *Min – Max* = minimum to maximum values.

References

Adams, D. C., & Otárola‐Castillo, E. (2013). Geomorph: An R package for the collection and analysis of geometric morphometric shape data. *Methods in Ecology and Evolution*, *4*(4), 393-399. https://doi.org/10.1111/2041-210X.12035

Bohn, M. (2014). UFC to introduce leg reach statistics for future events. *MMA Junkie*. Retrieved from https://mmajunkie.usatoday.com/2014/08/ufc-to-introduce-leg-reach-statistics-for-future-events

Bookstein, F. L. (1997). *Morphometric tools for landmark data*. Cambridge, UK: Cambridge University Press.

Caton, N. R., & Dixson, B. J. (2022a). Human third-party observers accurately track fighting skill and vigour along their unique paths to victory. *Scientific Reports*, *12*(1), 14841. https://doi.org/10.1038/s41598-022-19044-4

Caton, N. R., Hannan, J., & Dixson, B. J. (2022). Facial width‐to‐height ratio predicts fighting success: A direct replication and extension of Zilioli et al. (2014). *Aggressive Behavior*, *48*(5), 449-465. <https://doi.org/10.1002/ab.22027>

Caton, N. R., Pearson, S. G., & Dixson, B. J. (2022). Is facial structure an honest cue to real-world dominance and fighting ability in men? A pre-registered direct replication of. *Evolution and Human Behavior*, *43*(4), 314-324. <https://doi.org/10.1016/j.evolhumbehav.2022.04.002>

Hayes, A. F. (2013). Introduction to mediation, moderation, and conditional process analysis. A regression-based approach. New York, NY: Guilford Press.

Lane, S. M., & Briffa, M. (2020). Perceived and actual fighting ability: determinants of success by decision, knockout or submission in human combat sports. *Biology Letters*, *16*(10), 20200443. [http://dx.doi.org/10.1098/rsbl.2020.0443](about:blank)

Little, A. C., Třebický, V., Havlíček, J., Roberts, S. C., & Kleisner, K. (2015). Human perception of fighting ability: facial cues predict winners and losers in mixed martial arts fights. *Behavioral Ecology*, *26*, 1470-1475. https://doi.org/10.1093/beheco/arv089

Pugmire, L. (2017). UFC opens new $12-million performance institute in Las Vegas for fighters. *Los Angeles Times*. Retrieved from https://www.latimes.com/sports/boxing/la-sp-sn-ufc-performance-institute-dana-white-ari-emanuel-20170522-story.html

Rohlf, J. F. (2018a). tpsDig2 (Version 2.31) [Computer software]. New York, NY: Department of Ecology and Evolution, State University of New York at Stony Brook.

Třebický, V., Fialová, J., Kleisner, K., Roberts, S. C., Little, A. C., & Havlíček, J. (2015). Further evidence for links between facial width‐to‐height ratio and fighting success: Commentary on Zilioli et al. (2014). *Aggressive Behavior*, *41*, 331-334. https://doi.org/10.1002/ab.21559

Třebický, V., Havlíček, J., Roberts, S. C., Little, A. C., & Kleisner, K. (2013). Perceived aggressiveness predicts fighting performance in mixed-martial-arts fighters. *Psychological Science, 24*(9), 1664-1672. <https://doi.org/10.1177/0956797613477117>

Zilioli, S., Sell, A. N., Stirrat, M., Jagore, J., Vickerman, W., & Watson, N. V. (2015). Face of a fighter: Bizygomatic width as a cue of formidability. *Aggressive Behavior, 41*(4), 322-330. [https://doi.org/10.1002/ab.21544](about:blank)
